# TLR7 modulates extramedullary splenic erythropoiesis in P. yoelii NSM-infected mice through the regulation of iron metabolism of macrophages with IFN-γ

**DOI:** 10.3389/fimmu.2023.1123074

**Published:** 2023-04-27

**Authors:** Jiajie Li, Lin Liu, Junmin Xing, Dianhui Chen, Chao Fang, Feng Mo, Yumei Gong, Zhengrong Tan, Guikuan Liang, Wei Xiao, Shanni Tang, Haixia Wei, Shan Zhao, Hongyan Xie, Xingfei Pan, Xiaomao Yin, Jun Huang

**Affiliations:** ^1^ Department of Infectious Diseases, The Third Affiliated Hospital of Guangzhou Medical University, Guangzhou, China; ^2^ Sino-French Hoffmann Institute, Department of Basic Medical Science, Guangzhou Medical University, Guangzhou, China; ^3^ Department of Laboratory Medicine, Guangzhou Red Cross Hospital, Jinan University, Guangzhou, China; ^4^ Institute of Respiratory Health, The First Affiliated Hospital of Guangzhou Medical University, Guangzhou, China; ^5^ Department of Laboratory Medicine, Lecong Hospital, Foshan, China

**Keywords:** malaria, TLR7, extramedullary splenic erythropoiesis, IFN-γ, macrophages, iron metabolism

## Abstract

Splenomegaly is a prominent clinical manifestation of malaria and the causes remain incompletely clear. Anemia is induced in malaria and extramedullary splenic erythropoiesis is compensation for the loss of erythrocytes. However, the regulation of extramedullary splenic erythropoiesis in malaria is unknown. An inflammatory response could facilitate extramedullary splenic erythropoiesis in the settings of infection and inflammation. Here, when mice were infected with rodent parasites, *Plasmodium yoelii* NSM, TLR7 expression in splenocytes was increased. To explore the roles of TLR7 in splenic erythropoiesis, we infected wild-type and *TLR7* ^-/-^ C57BL/6 mice with *P. yoelii* NSM and found that the development of splenic erythroid progenitor cells was impeded in *TLR7* ^-/-^ mice. Contrarily, the treatment of the TLR7 agonist, R848, promoted extramedullary splenic erythropoiesis in wild-type infected mice, which highlights the implication of TLR7 on splenic erythropoiesis. Then, we found that TLR7 promoted the production of IFN-γ that could enhance phagocytosis of infected erythrocytes by RAW264.7. After phagocytosis of infected erythrocytes, the iron metabolism of RAW264.7 was upregulated, evidenced by higher iron content and expression of *Hmox1* and *Slc40a1*. Additionally, the neutralization of IFN-γ impeded the extramedullary splenic erythropoiesis modestly and reduced the iron accumulation in the spleen of infected mice. In conclusion, TLR7 promoted extramedullary splenic erythropoiesis in *P. yoelii* NSM-infected mice. TLR7 enhanced the production of IFN-γ, and IFN-γ promoted phagocytosis of infected erythrocytes and the iron metabolism of macrophages *in vitro*, which may be related to the regulation of extramedullary splenic erythropoiesis by TLR7.

## Introduction

Malaria, an epidemic caused by *Plasmodium* spp. parasites, is a serious health problem to be addressed currently (1). Anemia is one of the clinical manifestations of malaria, especially in young children (2). The malarial factors that contribute to anemia are intricate and multiple. For example, *Plasmodium* is an intraerythrocytic parasite that parasitizes red blood cells (RBCs), and destructs RBCs when schizont ruptures (1); intramedullary erythropoiesis is suppressed by inflammation (e.g., IL-6, TNF-α, and IFN-γ) (3, 4); RBCs clearance capacity in the spleen is upregulated in malaria because of spleen reorganization and splenomegaly (5); and reduced deformability make RBCs easier to be removed by the spleen (6, 7).

Similarly, anemia is usually observed in rodent malaria (8). Under the anemic stress, a great loss of RBCs and bone marrow erythropoiesis (steady state erythropoiesis) suppressed by inflammation, extramedullary splenic erythropoiesis is induced to maintain the erythroid hematopoiesis (9–11). In rodent model, malaria-induced anemia initiated extramedullary splenic erythropoiesis as well, and splenomegaly was observed simultaneously (8). It’s reported that splenic erythropoiesis was induced in the infection of *Salmonella*, and the population of erythroid cells increased most dramatically in the spleen, contributing to splenomegaly in mice (12). Whether erythroid cells expand most dramatically in the spleen and contribute to splenomegaly in malaria is unknown.

Extramedullary splenic erythropoiesis is distinct from bone marrow erythropoiesis, given that the signaling and progenitor cells stress erythropoiesis used are distinct from steady state erythropoiesis (11, 13). The BMP4-mediated signal is key for erythroid progenitor cell expansion and differentiation in the spleen (11), which could help to distinguish splenic burst forming unit-erythrocyte (BFU-E) from bone marrow BFU-E. Splenic BFU-E only requires erythropoietin (EPO) to form colonies, but bone marrow BFU-E forms colonies with the help of EPO and a second factor (11). They respond to an inflammation signal differentially. Inflammation supports extramedullary splenic erythropoiesis but suppresses bone marrow erythropoiesis (14–16). Pro-inflammatory cytokines, IL-1β and IFN-γ, promote the output of innate immune effector cells to address infection at the expenses of reduced bone marrow erythropoiesis (15, 17), however, TNF-α and IL-1β facilitated the expansion and differentiation of stress erythroid progenitor cells in the spleen (14). While TLRs activation shifts bone marrow hemopoiesis to innate immune effector cells (18), TLRs downstream signaling, Myd88, promotes splenic erythropoiesis in the condition of inflammation through increasing expression of GDF15 and BMP4 (bone morphogenetic protein 4) in the spleen (14). After the early stage of BFU-E and colony forming unit-erythrocyte (CFU-E), the erythroid progenitors subsequently undergo serial stages of differentiation including ProE, EryA, EryB, and EryC to mature (19). As the course of differentiation and maturation, the expression of CD71 decreases but the expression of Ter119 increases gradually (19). Based on the CD71 and Ter119 expressions, ProE, EryA, EryB, and EryC are distinguished and defined (19). Especially, ProE lacks Ter119, but EryC loses CD71. ProE, EryA, and EryB are immature erythroid progenitors. EryC refers to mature RBCs.

As a component of hemoglobins (20), iron is an essential element for erythropoiesis. Erythropoiesis in an adult human demands 20-25 mg of iron daily, however, the dietary iron requirement of an adult human is only 1-2 mg/day which can not meet the large demand of erythropoiesis (21). A large amount of iron is supplied by iron recycling of macrophages in the spleen where macrophages export Fe^2+^ into plasma by ferroportin (Fpn1 or Slc40a1) after phagocytosis of senescent erythrocytes and breakdown of hemoglobins (22). Once the Fe^2+^ is released, it is oxidized to ferric cation (Fe^3+^) by plasma ferroxidases, for example, copper-dependent ceruloplasmin, and then Fe^3+^ is bound to transferrin that allows iron to be transported around the body and utilized by erythroid progenitor cells after transferrin receptor recognition (22). In line with the iron homeostasis of humans, a primary source of iron in mice is iron recycling by splenic macrophages (23). Blocking the iron exporter of macrophages could reduce the serum iron level and the number of RBCs in mice (24). Iron recycling plays a critical role in iron homeostasis.

TLR7 is a member of the Toll-like receptors family, a type of pattern recognition receptors (PRRs). Its ligand is ssRNA, and ssRNA from *Plasmodium* in the endosome could activate TLR7 signaling in macrophages and dendritic cells (DCs) after the uptake of parasites or parasite components (25). In lethal *Plasmodium yoelii* YM infection, it’s plasmacytoid cells (pDCs) that sense the infection through TLR7 and produce a large amount of IFN-α and IFN-β *via* Myd88-dependent IFN regulatory factor (IRF) 7 transcription factor-mediated type I IFN signaling in the first 24 hours post infection (26). Subsequently, IFN-α and IFN-β trigger the anti*-*malaria immunity that requires macrophages and cDCs (26). Parallelly, in the infection of *Plasmodium chabaudi*, TLR7 is the primary Myd88-dependent sensor at 24 hours post infection, promoting IFN-α and IFN-β production and a cytokine response (IL-10, TNF, IL-12, and IFN-γ) (27). However, pDCs produce type I IFN *via* TLR7/MyD88 in the bone marrow, and type I IFN activates inflammatory monocytes and NK cells in the blood, which is a lethal factor in severe malaria (*Plasmodium yoelii* YM infection) (28). In malaria, TLR7 recognizes RNA from *Plasmodium*, induces the robust production of type I IFN *via* the downstream signaling, and further upregulates the production of IL-12 and IFN-γ to initiate the pro-inflammatory cytokines response, which is an important mechanism of anti-*Plasmodium* infection during the early stage. It’s reported that IFN-γ can enhance erythrophagocytosis by macrophages (29, 30), and TLRs downstream signaling, Myd88, can reduce SIRPα of macrophages to inhibit the axis of SIRPα-CD47 and stimulate erythrophagocytosis as well (14). Taken together, a hypothesis that TLR7 promotes iron recycling that supports erythropoiesis through enhancing erythrophagocytosis by macrophages was proposed.

No evidence suggests the regulation of malaria-induced splenic erythropoiesis by TLR7. In our study, we attempted to investigate the effect of TLR7 on malaria-induced splenic erythropoiesis in *P. yoelii*-infected C57BL/6 mice. Moreover, the mechanism through which TLR7 regulates malaria-induced splenic erythropoiesis was explored as well. This study may give us an insight into the relationship between TLR7-induced inflammatory response and malaria-induced splenic erythropoiesis and promote the development of a therapeutical strategy based on the target of TLR7.

## Materials and methods

### Mice

Wild-type C57BL/6 mice were purchased from Guangdong Zhiyuan Biomedical Technology Co., Ltd., and TLR7^-/-^ C57BL/6 mice (B6.129S1-Tlr7^tm1Fl^v/J) were purchased from The Jackson Laboratory. All mice were fed in a specific pathogen-free micro-environment at the Experimental Animal Centre, Guangzhou Medical University. The animal study was approved by the Institutional Animal Care and Use Committee of Guangzhou Medical University (2012–11).

### Infection

The *Plasmodium* strain used is *Plasmodium yoelii* NSM (*P. yoelii* NSM) and infected red blood cells (iRBCs) were preserved at -80°C. The frozen iRBCs were thawed in a 37°C water bath and injected into 4-week-old mice intraperitoneally and the fresh iRBCs were injected into 6 or 8-week-old experimental female mice intraperitoneally (1×10^6^ iRBCs per mouse). The uninfected and infected mice were sacrificed by cervical dislocation under anesthesia.

### Treatment of R848 or IFN-γ

The scheme of R848 treatment was that mice were injected on the day before infection and 2, 5, 8, and 11 days after infection with 100 μg of R848 (Sigma) respectively. For *in vivo* blocking of IFN-γ, mice were injected with 100μg of anti-mouse IFN-γ (XMG1.2, BioX Cell) at 3, 6, 9, and 12 days after infection respectively.

### Detection of serum and hematologic parameters

Blood was collected from the orbit and placed into EDTA-containing tubes and serum was isolated from the blood that was preserved in the coagulant-containing tubes. The blood and serum samples were detected in the Laboratory Department of the Sixth People’s Hospital of Panyu District (Guangdong Province, China). The indexes of Hematology were detected by the Sysmex XN hematology analyzer, and the indexes of iron metabolism were detected by the Beckman Coulter AU5800 analyzer.

### Isolation of splenocytes and bone marrow cells and flow cytometry

The spleen harvested from the mouse abdomen was ground by a sterile syringe piston and filtered with a 100-μm cell strainer. Bone marrow was obtained by flushing the femurs with RPMI 1640 medium (Gibco). Then, the cell suspension was collected and RBCs were removed by RBC lysis buffer. The remaining cells were washed by HBSS (Hank’s balanced salt solution) and resuspended in 1640 medium supplemented with 10% fetal bovine serum (FBS) (Gibco), penicillin (50 U/mL, Gibco), and streptomycin (50 mg/mL, Gibco) finally. The cells were washed with phosphate-buffered saline (PBS) and blocked with CD16/CD32 antibodies (Biolegend) for 10 minutes at 4°C and then stained with fluorescent antibodies (Biolegend) including PerCP/Cyanine5.5 anti-mouse CD71(RI7217), FITC anti-mouse TER-119/Erythroid Cells (TER-119), Alexa Fluor^®^ 700 anti-mouse CD45 (I3/2.3), PE/Cyanine7 anti-mouse CD45 (I3/2.3), PE/Cyanine7 anti-mouse/human CD11b (M1/70), APC/Cyanine7 anti-mouse F4/80 (BM8), PE anti-mouse TLR7 (A94B10), PE anti-mouse CD3ϵ (145-2C11), PerCP/Cyanine5.5 anti-mouse CD19 (6D5), PE/Cyanine7 anti-mouse NK-1.1 (PK136), APC anti-mouse IFN-γ (XMG1.2). Detection of CD45^+^ Ter119^-^ cells and CD45^-^ Ter119^+^ cells was performed before the lysis of RBCs. To detect intracellular cytokine IFN-γ, the cells were first stimulated with PMA (20 ng/ml, Sigma) and ionomycin (1 µg/ml, Sigma) at 37°C, 5% CO_2_ for 5 h and incubated with brefeldin A (10 µg/ml, Sigma) for final 4 h to inhibit cytokines secretion. After that, the cells were stained with surface markers and fixed with fixation and permeabilization solution (BD Biosciences) for 20 min at 4°C in the dark and then permeabilized for 30 minutes at 4°C in saponin-containing buffer (BD Biosciences). IFN-γ fluorescent antibody was added to stain cells after washing with saponin-containing buffer. Annexin V-FITC (Lianke Bio) was added and incubated with cells for 10 min at room temperature. Cells were detected by flow cytometry (Beckman Coulter) and results were analyzed with the software CytoExpert 2.3 (Beckman Coulter) or FlowJo v10 (Flow Jo, LLC).

### Quantitative real-time PCR

Splenocytes and RAW264.7 cells were preserved in TRIzol reagent (Invitrogen) for RNA extraction by using chloroform, isopropanol, and 75% ethanol. 1 μg of total RNA was transcribed to cDNA by using HiScript^®^ III RT SuperMix for qPCR (Vazyme) and quantitative real-time PCR (qRT-PCR) was performed by using ChamQ Universal SYBR qPCR Master Mix (Vazyme) and measured by CFX96 Real-Time PCR Detection System (Bio-Rad). Data were normalized to housekeeping gene *β-actin*. The primer sequences were: *IFNg*, CAAGTGGCATAGATGTGGAAGA (forward) and GTTGACCTCAAACTTGGCAATAC (reverse); *Hmox1*, TGATGGCTTCCTTGTACCATATC (forward) and CTCGTGGAGACGCTTTACATAG (reverse); *Spic*, CCAGAGCCCTGAGGAATTATG (forward) and TGCCGTGAGCATAGTGATTT (reverse); *Slc40a1*, TGGAACTCTATGGAAACAGCCT (forward) and TGGCATTCTTATCCACCCAGT (reverse); *β-actin*, CCGTAAAGACCTCTATGCCAAC (forward) and GGGTGTAAAACGCAGCTCAGTA (reverse).

### Single-cell RNA sequencing

Splenocytes from uninfected and infected mice were obtained according to the protocol mentioned above and CD45^+^ cells were sorted by FACS (Beckman MoFlo). After the viability detection and cell count, the RNA expression profile in each cell was detected by 10x Genomics Chromium Single Cell 3’s platform in YUANXIN Biotechnology CO., LTD (Guangzhou, China). First, the single cell was barcoded and underwent reverse transcription in oil droplets for preparation of the cDNA library by the Chromium Single Cell 3′Library & Gel Bead Kit v3. Then, libraries were sequenced on 8 lanes of Illumina Nova seq6000 using paired-end 150 bp.

All data processing and analysis were performed in YUANXIN Biotechnology CO., LTD. We used CellRanger (version 3.1.0), aligned reads on the GRCm38 reference genome for mouse and generated unique molecular identifier gene expression profiles for every single cell under standard sequencing quality threshold (default parameters). Low-quality cells were removed for downstream analysis when they met the following criteria for retaining cells: (1) ≥50,000 sequence reads; (2) ≥40% of reads uniquely aligned to the genome; (3) ≥40% of these reads mapping to RefSeq annotated exons. For further filtration, undesirable cells were removed under the criteria: (1) expressed less than 200 and more than 6000; (2) mitochondrial genome transcript was higher than 10%; (3) genes expressed in less than 3 cells; (4) more than 50000 UMI counts. Finally, 13790 cells and 18795 genes remained in data from the uninfected mouse, and 11829 cells and 19333 genes remained in data from the infected mouse.

The final filtered gene expression data matrix was normalized by using the “NormalizeData” function with default setting and 4000 highly variable genes were chosen through the “FindVariableGenes” function to be centered and scaled *via* the “ScaleData” function. Then, principal component analysis (PCA) was performed on the 4000 genes by the “RunPCA” function, and dimensional reduction was performed through Canonical correlation analysis (CCA) in Seurat. The “FindClusters” function was used to cluster cells and the clustered cells were shown in a uniform manifold approximation and projection (UMAP) plot.

The “FindMarkers” function of Seurat was used to identify differentially expressed genes (DEGs) by using “Wilcox” test methods and Bonferroni correction. Significant DEGs with P value (p_val) ≤ 0.05 and log processed average fold change (avg_log2FC) ≥ 0.25 were further analyzed and visualized. GO analysis and KEGG pathway enrichment analysis of these significant DEGs were performed by using the clusterProfiler package.

### Isolation and lysis of iRBCs

IRBCs were isolated from the blood that was collected from infected mice with 30% or higher parasitemia. Briefly, blood was centrifuged at 2500 rpm, 4°C for 5 minutes to remove the plasma and the remaining RBCs were washed with PBS. Then, the whole RBCs were resuspended in 1 ml PBS and overlayed onto 2 ml percoll gradient (50% and 70%) in a 15 ml centrifuge tube to centrifuge at 300 g, 4°C, ± 2 acceleration for 30 minutes. IRBCs were collected from the interface and washed with PBS. Lysates of iRBCs were harvested by three freeze-thaw cycles in liquid nitrogen and a 37°C water bath.

### Cells culture and stimulation

RAW264.7 cells were cultured in DMEM medium supplemented with 10% FBS (Gibco), penicillin (50 U/mL, Gibco), and streptomycin (50 mg/mL, Gibco) at 37°C and 5% CO_2_. 2×10^5^ cells were inoculated in 48 well plates on the day before stimulation, and cells were stimulated with PBS, R848 (2 µg/ml, Sigma), mouse recombinant IFN-γ (200 ng/ml, Novoprotein), iRBCs (iRBCs: cells = 3: 1), R848 plus iRBCs, IFN-γ plus iRBCs respectively for 20 h.

### ELISA

According to the manufacturer’s instructions, IFN-γ levels of serum and supernatant were measured by using Mouse IFN-γ ELISA Set (BD), serum erythroferrone was measured by using Mouse Erythroferrone ELISA Kit (Bio-Swamp), and serum hepcidin was measured by using Mouse Hepcidin ELISA Kit (Sangon Biotech). Results were read at 450 nm by using a microplate reader (Moder ELX- 800, BioTek).

### *In vitro* phagocytosis assay

Uninfected RBCs (uRBCs) and iRBCs were labeled with CellTrace CFSE (Invitrogen) according to the manufacturer’s instructions. RAW264.7 cells or splenocytes were cocultured with labeled uRBCs or iRBCs for 5 h and were detected by flow cytometry.

### Fe^2+^, lipid peroxidation, and reactive oxygen species detection

For detection of Fe^2+^ in cells, splenocytes or RAW264.7 cells were incubated with FerroOrange (1µM, DOJINDO) following either infection *in vivo* or stimulation *in vitro* at 37°C in dark. Cells were washed twice with PBS and then stained with surface markers if necessary. For detection of lipid peroxidation in cells, RAW264.7 cells were incubated with C11-BODIPY 581/591 (2 µM, Invitrogen) following stimulation *in vitro* at 37°C in dark, then, cells were washed twice with PBS containing 1% FBS. To measure reactive oxygen species (ROS) level in cells, RAW264.7 cells were incubated with DCFH-DA (10 µM, Beyotime) following stimulation *in vitro* at 37°C in dark, then, cells were washed twice with PBS containing 1% FBS. Cells were immediately detected by flow cytometry at last.

### IRBCs detection by flow cytometry

Cells collected from the spleen, bone marrow, or blood were washed twice with PBS containing 1% BSA and stained with CD45, TER-119, and CD71 fluorescent antibodies. Then, cells were incubated with Hoechst 34580 (0.5 µg/ml, BD) at room temperature in dark for 30 min to be measured by flow cytometry. In CD45^-^ Ter119^+^ cells, CD71^-^ indicated mature and enucleated erythroid cells, and CD71^+^ indicated less mature erythroid progenitor cells that may contain DNA molecules. Hoechst, a DNA dye with cell- permeability, could stain parasite and erythroblast DNA. In CD45^-^ Ter119^+^ CD71^-^ population, uninfected mature erythroid cells without DNA molecules are Hoechst^neg^ (CD45^-^ Ter119^+^ CD71^-^ Hoechst^neg^), infected mature erythroid cells that only contain parasite DNA are Hoechst^pos^ (CD45^-^ Ter119^+^ CD71^-^ Hoechst^pos^). In CD45^-^ Ter119^+^ CD71^+^ population, uninfected and relatively mature erythroid progenitor cells without DNA molecules are Hoechst^neg^ (CD45^-^ Ter119^+^ CD71^+^ Hoechst^neg^), uninfected and less mature erythroid progenitor cells that only contain erythroblast DNA are Hoechst^low^ (CD45^-^ Ter119^+^ CD71^+^ Hoechst^low^), infected and less mature erythroid progenitor cells that contain erythroblast and parasite DNA are Hoechst^hi^ (CD45^-^ Ter119^+^ CD71^+^ Hoechst^hi^).

### Perls’ Prussian blue staining

8 µm sections of the spleen were washed with distilled water before staining. The sections were stained with Perls’ Prussian Blue for 40 min and then stained with Neutral Red for 2 min. After staining, hemosiderin or trivalent iron (Fe^3+^) is blue, and the nucleus and other tissues are red. Stained sections were scanned by a Digital Section Scanner (Leica). The percentage of blue area in the section is calculated by using ImageJ (V1.8.0).

### Statistical analysis

Unpaired t-tests test was used for comparisons between the two groups with equal variance and normal distributions and ANOVA with Sidak multiple comparisons test was used for comparisons between more than two groups with normal distributions and equal variance, unless differently specified. When various groups have unequal variance, ANOVA with Tamhane T2 was used to analyze. Statistical differences were defined as P < 0.05. All data were analyzed in GraphPad Prism (v9).

## Results

### Accumulation of erythroid cells in the spleen is a key factor for splenomegaly in *P. yoelii* NSM-infected mice

**Figure 1:**
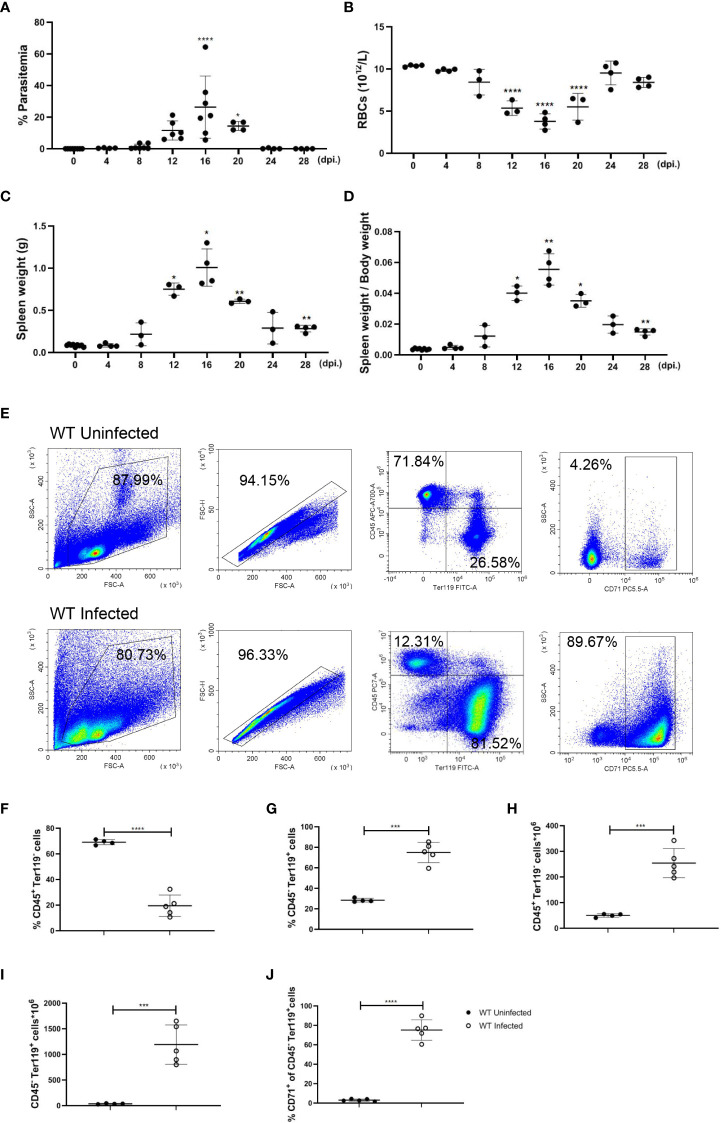
P*. yoelii* NSM infection results in the accumulation of erythroid cells in the spleen of C57BL/6 mice. **(A)** Parasitemia, **(B)** the absolute number of RBCs in peripheral blood, **(C)** spleen weight, and **(D)** the ratio of spleen weight over body weight of wild-type mice were measured for 28 days after infection. **(E)** Representative pseudocolor plots of CD45^+^ Ter119^-^ cells, CD45^-^ Ter119^+^ cells, and CD45^-^ Ter119^+^ CD71^+^ cells in the spleen. **(F)** Percentage and **(H)** the absolute number of CD45^+^ Ter119^-^ cells in spleens from wild-type uninfected and infected mice were analyzed at 16 dpi. **(G)** Percentage and **(I)** the absolute number of CD45^-^ Ter119^+^ cells in spleens from wild-type uninfected and infected mice were analyzed at 16 dpi. **(J)** The percentage of CD45^-^ Ter119^+^ CD71^+^ cells in spleens from wild-type uninfected and infected mice were analyzed at 16 dpi. **(A-D)** n=3-7 mice per group; **(F-J)** n=4-5 mice per group; Data shown as mean ± SEM are representative of three independent experiments; *P < 0.05, **P < 0.01,***P < 0.001, ****P < 0.0001, ANOVA with Sidak or Tamhane T2 multiple comparison test or unpaired t-test.

Our previous finding indicates that infection of *P. yoelii* NSM could stimulate a T cell-mediated inflammatory response in C57BL/6 mice, and unlike *P. yoelii* XL, *P. yoelii* NSM usually induces a non-lethal infection in C57BL/6 mice (31), which is an appropriate model for an investigation into malaria-induced splenic erythropoiesis. After being infected with *P. yoelii* NSM, parasitemia, the number of RBCs, spleen weight, and body weight were monitored in infected mice for 28 days, showing the most serious condition at 12-20 day post-infection (dpi.). At 16 dpi., while parasitemia (26.36%), spleen weight (1.01 g), and the ratio of spleen weight over body weight (0.0555), were the highest relative to uninfected status the number of RBCs in the blood (3.775×10^12^/L) was the lowest relative to uninfected status (**Figures 1A–D**). To examine changes in splenic cellularity during the serious period, flow cytometry was used to distinguish immune cells (CD45^+^ Ter119^-^) and more mature developing erythroid cells (CD45^-^ Ter119^+^) in the spleen (**Figure 1E**), and the results showed that the percentage of splenic CD45^+^ Ter119^-^ cells decreased but the percentage of splenic CD45^-^ Ter119^+^ cells increased at 16 dpi. (**Figures 1F, G**). The absolute number of splenic CD45^+^ Ter119^-^ cells and splenic CD45^-^ Ter119^+^ cells increased dramatically (**Figures 1H, I**). CD71, a transferrin receptor expressed by erythroid progenitor cells, declines as the development of erythroid progenitor cells and is lost on the surface of mature erythrocytes (32). About 70% of splenic CD45^-^ Ter119^+^ cells in spleens that were observed in infected mice were marked with CD71 (**Figure 1J**). These findings suggested that erythroid cells accumulated in the spleen of *P. yoelii* NSM-infected mice, which is likely a key contributor to splenomegaly, and that the accumulation of erythroid cells in the spleen is a potential outcome of extramedullary splenic erythropoiesis. Therefore, 12-20 dpi is an appropriate time point to investigate the regulation of the extramedullary splenic erythropoiesis, and the following experiments were performed at this time point.

### Expression of TLR7 is increased and modulates extramedullary splenic erythropoiesis in *P. yoelii* NSM-infected mice

**Figure 2:**
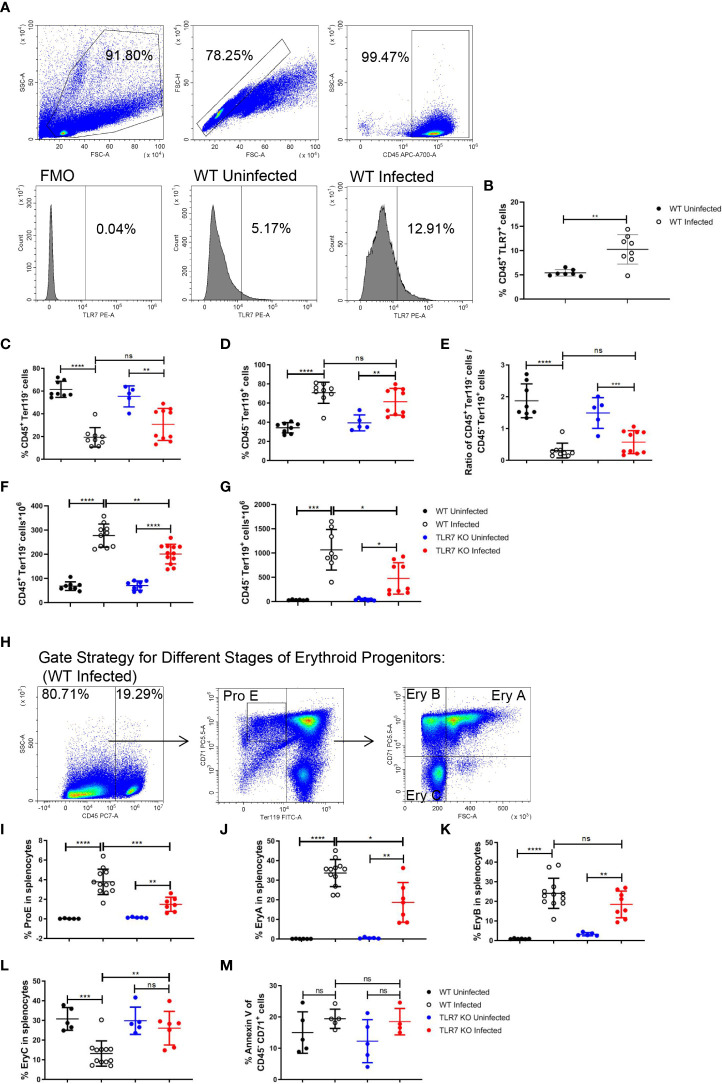
TLR7 modulates extramedullary splenic erythropoiesis in *P. yoelii* NSM-infected C57BL/6 mice. **(A)** Representative pseudocolor plots show the gating strategy for splenic CD45^+^ cells and representative histograms show the TLR7 expression level of wild-type uninfected and infected mice. **(B)** The percentage of CD45^+^ TLR7^+^ cells in spleens from wild-type uninfected and infected mice were analyzed at 16 dpi. **(C)** Percentage and **(F)** the absolute number of CD45^+^ Ter119^-^ cells in spleens from wild-type uninfected and infected, *TLR7* ^-/-^ uninfected and infected mice were analyzed at 16 dpi. **(D)** Percentage and **(G)** the absolute number of CD45^-^ Ter119+ cells in spleens from wild-type uninfected and infected, *TLR7* ^-/-^ uninfected and infected mice were analyzed at 16 dpi. **(E)** The ratio of the percentage of CD45^+^ Ter119^-^ cells over the percentage of CD45^-^ Ter119^+^ cells of wild-type uninfected and infected, *TLR7* ^-/-^ uninfected and infected mice were analyzed at 16 dpi. **(H)** Gating strategy of four subsets of erythroid progenitors including ProE, EryA, EryB, and EryC. **(I-L)** Percentages of ProE, EryA, EryB, or EryC in splenocytes from wild-type uninfected and infected, *TLR7* ^-/-^ uninfected and infected mice were analyzed at 16 dpi. **(M)** The percentage of Annexin V^+^ CD45^-^ CD71^+^ cells in spleens from wild-type uninfected and infected, *TLR7* ^-/-^ uninfected and infected mice were analyzed at 16 dpi. **(B)** n=7-8 mice per group; **(C-G)** n=5-12 mice per group; **(I-L)** n=5-12 mice per group; **(M)** n=4-5 mice per group; Data shown as mean ± SEM are representative of three independent experiments; *P < 0.05, **P < 0.01,***P < 0.001, ****P < 0.0001, ns, not significant, *P >*0.05; ANOVA with Sidak multiple comparison test or unpaired t-test.

As inflammation is a critical factor in stress erythropoiesis, the increased level of TLR7 expression in splenic CD45^+^ cells after the infection (**Figures 2A, B**) triggers our interest in studying the role of the innate immune response induced by TLR7 in extramedullary splenic erythropoiesis. Wild-type and *TLR7* ^-/-^ mice were infected with *P. yoelii* NSM and we found that the parasitemia of *TLR7* ^-/-^ infected mice seemed more severe (**Supplementary Figure S1A**). Meanwhile, *TLR7* ^-/-^ infected mice exhibited more severe malaria-induced anemia. The amount of RBC in the blood was reduced more dramatically in *TLR7* ^-/-^ infected mice, although there was no difference in hemoglobin and hematocrit between these two groups of infected mice (**Supplementary Figures S1B-D**). Besides, mean corpuscular volume and red blood cell distribution width were higher but mean corpuscular hemoglobin concentration was lower in *TLR7* ^-/-^ infected mice (**Supplementary Figures S1E-H**). Similar to wild-type mice, the percentage of splenic CD45^+^ Ter119^-^ cells was decreased and the percentage of splenic CD45^-^ Ter119^+^ cells was increased in *TLR7* ^-/-^ mice (**Figures 2C-E**). The absolute number of CD45^+^ Ter119^-^ cells or CD45^-^ Ter119^+^ cells in the spleen increased, but the absolute number of CD45^-^ Ter119^+^ cells was larger than CD45^+^ Ter119^-^ cells (**Figures 2F, G**), which was consistent with that the expansion of erythroid cells primarily contributed to splenomegaly. The absolute number of CD45^-^ Ter119^+^ cells in *TLR7* ^-/-^ mice was less than in wild-type mice (**Figure 2G**). For understanding which stage of erythroid progenitor cells was affected, we distinguished the serial differentiation of ProE (CD71^high^ Ter119^low^), EryA (CD71^high^ Ter119^high^ FSC^high^), EryB (CD71^high^ Ter119^high^ FSC^low^) and EryC (CD71^low^ Ter119^high^ FSC^low^) based on the expression level of Ter119 and CD71 and cell volume (**Figure 2H**, **Supplementary Figure S2**) (19, 32). After the infection, the percentage of splenic ProE or splenic EryA in wild-type mice was higher than in *TLR7* ^-/-^ mice (**Figures 2I, J**), however, the percentage of splenic EryB in wild-type mice and *TLR7* ^-/-^ mice were similar (**Figure 2K**), resulting in a lower percentage of splenic EryC in wild-type mice than in *TLR7* ^-/-^ mice (**Figure 2L**).

Similarly, the erythropoiesis response was stimulated in bone marrow (BM) after the infection in our model. The percentage of BM CD45^+^ Ter119^-^ cells was decreased and the percentage of BM CD45^-^ Ter119^+^ cells was increased after the infection (**Supplementary Figures S3A-C**). The percentage of BM ProE or BM EryA increased after the infection (**Supplementary Figures S3D, E**). The percentage of BM EryB was not altered after the infection (**Supplementary Figure S3F**). The percentage of BM EryC was reduced after the infection (**Supplementary Figure S3G**). Nevertheless, we did not observe any difference in bone marrow erythropoiesis response between wild-type and *TLR7* ^-/-^ mice following the infection. PS exposure at the cell surface of RBCs could be induced by *Plasmodium* and facilitate phagocytosis of RBCs by macrophages that display a series of receptors for PS, which is considered to be a secondary mechanism of removal of RBCs (23, 33, 34). An increase in the percentage of annexin V^+^ in BM CD45^-^ CD71^+^ cells (erythroid progenitor cells) was observed after the infection (**Supplementary Figure S3H**), which could not be observed in the spleen (**Figure 2M**), indicating that splenic erythropoiesis is more efficient than bone marrow erythropoiesis. Additionally, the expression of TLR7 could not be detected in CD45^-^ cells (**Supplementary Figure S3I**). These demonstrated that TLR7 modulates splenic erythropoiesis but not bone marrow erythropoiesis through an indirect way after the infection.

Wild-type infected mice were treated with R848 to highlight the impact of TLR7 on splenic erythropoiesis. Treatment of R848 did not affect the percentage of splenic CD45^+^ Ter119^-^ cells or splenic CD45^-^ Ter119^+^ cells (**Supplementary Figures S4A-C**). Though treatment of R848 did not regulate the absolute number of splenic CD45^+^ Ter119^-^ cells, it significantly increased the absolute number of splenic CD45^-^ Ter119^+^ cells (**Supplementary Figures S4D, E**). Moreover, the percentage of ProE was increased by treatment of R848 (**Supplementary Figure S4F**), but EryA, EryB, and EryC were not the targets regulated by R848 (**Supplementary Figures S4G-I**).

The spleen is responsible for the clearance of iRBCs (35). To investigate whether iRBCs that were sequestered in the spleen contributed to the increased percentage of CD45^-^ Ter119^+^ cells, the percentage of infected CD45^-^ Ter119^+^ cells were measured according to Hoechst 34580 fluorescence in cells (**Supplementary Figure S5A**) (36, 37). Most of the infected CD45^-^ Ter119^+^ cells in the spleen or bone marrow were marked with CD71, which could not be observed in the blood (**Supplementary Figure S5A**). Infected CD45^-^ Ter119^+^ cells were just a small portion of erythroid cells in the spleen or bone marrow, although they were higher than the percentage in the blood (**Supplementary Figures S5B, C**). It suggested that the erythroid progenitor cells in the spleen were newly formed. Therefore, extramedullary splenic erythropoiesis was induced after the infection of *P. yoelii* NSM.

### TLR7 induces the production of IFN-γ in splenocytes of *P. yoelii* NSM-infected mice

**Figure 3:**
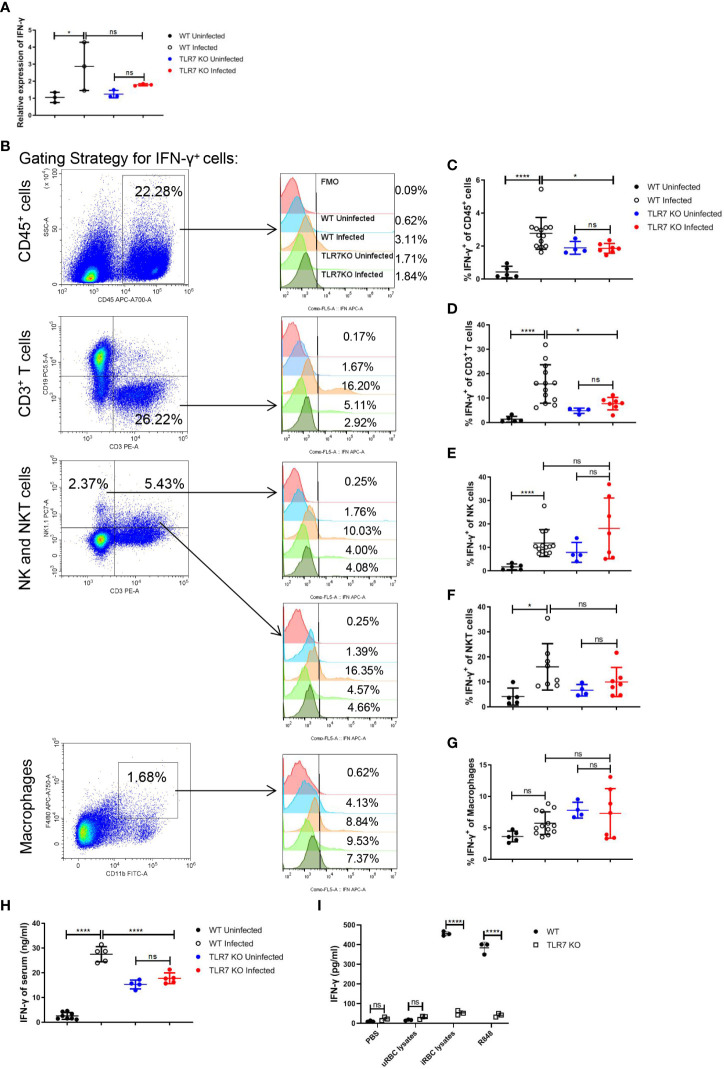
The knockout of *TLR7* impairs IFN-γ production in the spleen of *P. yoelii* NSM-infected C57BL/6 mice. **(A)** Relative *IFN-γ* mRNA levels in splenocytes from wild-type uninfected and infected, *TLR7* ^-/-^ uninfected and infected mice were measured by qPCR at 16 dpi. **(B)** Representative pseudocolor plots show the gating strategy for splenic CD45^+^ cells, T cells (CD45^+^, CD3^+^), NK (CD45^+^, CD3^-^, NK1.1^+^) cells, NKT (CD45^+^, CD3^+^, NK1.1^+^) cells and macrophages (CD45^+^, CD11b^+^, F4/80^+^) and representative histograms show their IFN-γ expression level respectively. **(C-G)** IFN-γ expression levels of splenic CD45^+^ cells, T cells, NK cells, NKT cells, and macrophages from wild-type uninfected and infected, *TLR7* ^-/-^ uninfected and infected mice were analyzed respectively at 16dpi. **(H)** IFN-γ levels of serum from wild-type uninfected and infected, *TLR7* ^-/-^ uninfected and infected mice were detected by ELISA at 16dpi. **(I)** Splenocytes from wild-type and *TLR7* ^-/-^ uninfected mice were stimulated with PBS, uRBC lysates, iRBC lysates, and R848 respectively *in vitro* and the supernatant was collected after 48 hours to detect IFN-γ level by ELISA. **(A)** n=3 mice per group; **(C-G)** n=4-13 mice per group; **(H)** n=4-8 mice per group; **(I)** n=3 samples per group; Data shown as mean ± SEM are representative of three independent experiments; *P < 0.05, ****P < 0.0001, ns, not significant, *P >*0.05; ANOVA with Sidak or Tamhane T2 multiple comparisons test or unpaired t-test.

It’s reported that the TLR7 activation could stimulate Th1 cell response, inducing a higher percentage of IFN-γ^+^ CD4^+^ T cells in the spleen at 15 dpi. in *P. chabaudi*-infected mice (38). Given that, we hypothesized that the production of IFN-γ could be influenced by TLR7 signaling after *Plasmodium* infection. In our experiments, an increase in *IFNg* mRNA in splenocytes by *Plasmodium* infection was abolished by the knockout of *TLR7* (**Figure 3A**). The production of IFN-γ in different splenic populations including immune cells (CD45^+^), T cells (CD45^+^, CD3^+^), NK cells (CD45^+^, CD3^-^, NK1.1^+^), NKT cells (CD45^+^, CD3^+^, NK1.1^+^) and macrophages (CD45^+^, CD11b^+^, F4/80^+^) were measured by flow cytometry (**Figure 3B**). *TLR7* ^-/-^ mice displayed a lower level of the percentage of splenic CD45^+^ IFN-γ^+^ cells than wild-type mice after the infection, which was a consequence of reduced production of IFN-γ in splenic T cells (**Figures 3C-G**). Additionally, wild-type infected mice exhibited a higher level of serum IFN-γ than *TLR7* ^-/-^ infected mice (**Figure 3H**).To demonstrate that iRBCs components can activate TLR7 to induce the production of IFN-γ, uRBCs lysates, iRBCs lysates, and R848 that was positive control were used to stimulate splenocytes from wild-type and *TLR7* ^-/-^ uninfected mice for 48 h *in vitro* respectively. R848 and iRBCs lysates only stimulated splenocytes from wild-type mice to produce a great amount of IFN-γ (**Figure 3I**). All of these indicated that IFN-γ response is a downstream event of TLR7 activation.

### Splenic macrophages respond to IFN-γ and upregulate iron metabolism after *P. yoelii* NSM infection

**Figure 4:**
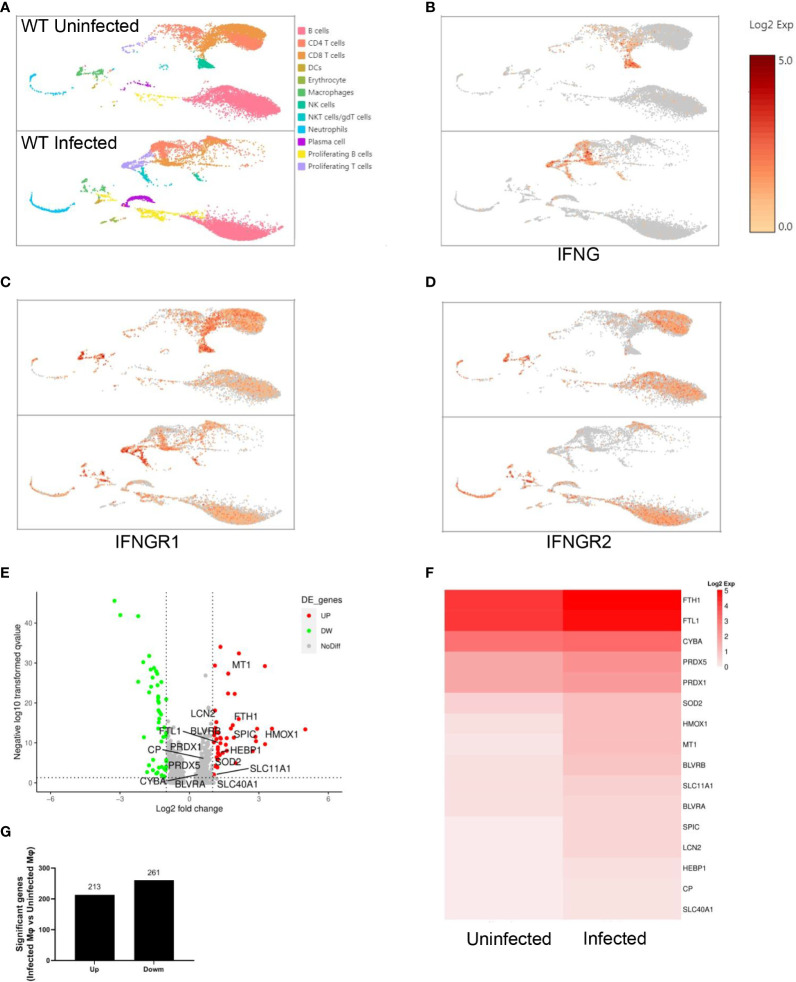
Splenic macrophages respond to IFN-γ after the infection of *P. yoelii* NSM. Single-cell RNA sequencing of splenic CD45^+^ cells from wild-type uninfected and infected mice was performed at 16 dpi. Splenic CD45^+^ cells were sorted by FACS and the expression profile in single cells was detected using single-cell RNA sequencing (10× Genomics Chromium system). **(A)** UMAP of splenic CD45^+^ cells. Splenic CD45^+^ cells were classified into 12 clusters based on the properties of the RNA expression profile in each cell. Every cluster was colored and labeled. **(B-D)** Expression levels of IFNG, IFNGR1, and IFNGR2. **(E-G)** Differential gene expression in macrophages from wild-type infected mice compared with macrophages from wild-type uninfected mice. All differentially expressed genes were distributed in **(E)** volcano plot according to the adjusted p values (-log10(padj)) and fold changes (log2 fold change). Red dots indicate the up-regulated genes (adjusted p values < 0.05 and fold changes ≥ 1.5); Green dots indicate the down-regulated genes (adjusted p values < 0.05 and fold changes ≤ -1.5); Grey dots indicate the non-significant genes (adjusted p values > 0.05 or |fold changes| < 1.5). The labeled genes were involved in iron metabolism. **(G)** The number of up-regulated genes and down-regulated genes. **(F)** The heatmap shows the expression level of genes involved in iron metabolism in macrophages from wild-type uninfected and infected mice.

**Figure 5:**
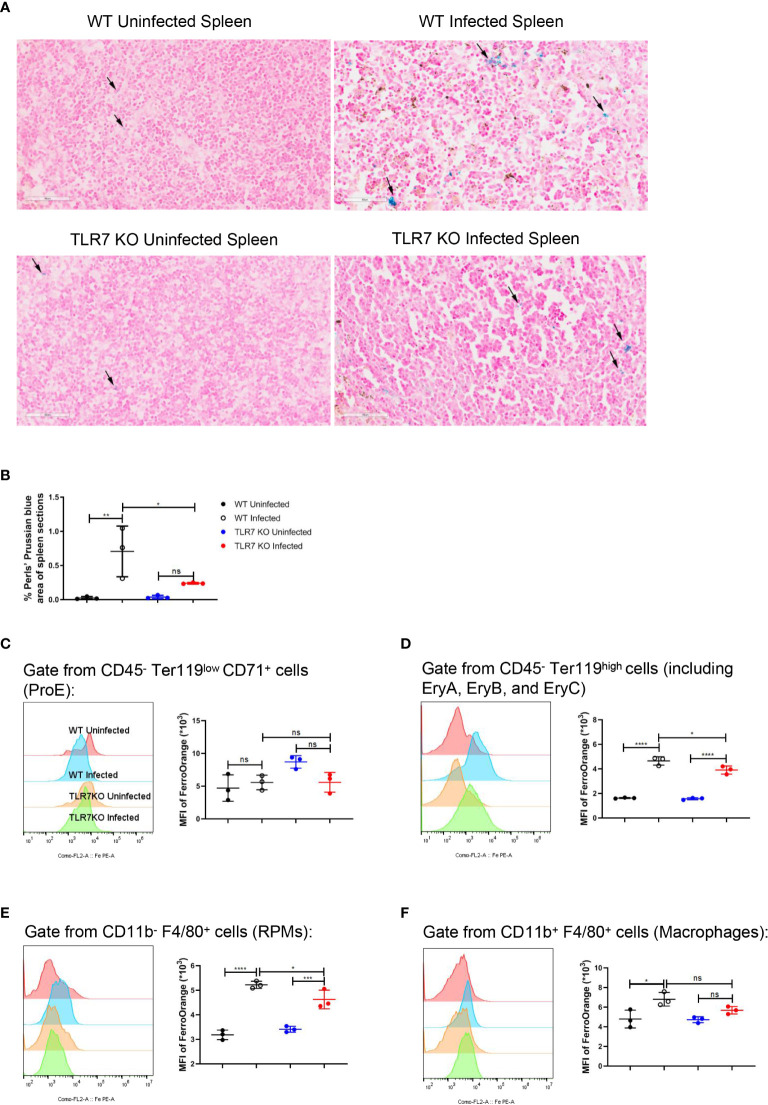
The knockout of TLR7 reduces the accumulation of iron in the spleen from *P. yoelii* NSM-infected C57BL/6 mice. **(A)** Perls’ Prussian blue staining in sections of spleens from wild-type uninfected and infected, *TLR7* ^-/-^ uninfected and infected mice was performed at 16 dpi. **(B)** Percentages of Perls’ Prussian blue area in the spleen sections from wild-type uninfected, wild-type infected, *TLR7* ^-/-^ uninfected, and *TLR7* ^-/-^ infected mice were calculated and analyzed at 16 dpi. The mean fluorescence intensity of FerroOrange was detected in ProE **(C)**, the population including EryA, EryB, and EryC **(D)**, RPMs **(E)**, and splenic macrophages **(F)** at 16 dpi. **(B)** n=3 mice per group. **(C-F)** n=3 mice per group. Data shown as mean ± SEM are representative of two or three independent experiments; *P < 0.05, **P < 0.01,***P < 0.001, ****P < 0.0001, ns, not significant, *P >*0.05; ANOVA with Sidak multiple comparisons test.

Single-cell RNA sequencing of splenic CD45^+^ cells from wild-type uninfected and infected mice was performed to find the mediators that IFN-γ utilizes to regulate extramedullary splenic erythropoiesis. Splenic CD45^+^ cells were classified into 12 clusters including B cells, CD4^+^ T cells, CD8^+^ T cells, DCs, erythrocytes, macrophages, NK cells, γδT cells/NKT cells, neutrophils, plasma cells, proliferating B cells and proliferating T cells and all clusters were displayed on UMAP plot (**Figure 4A**). Consistent with the results detected by flow cytometry (**Figure 3B**), high expression of IFN-γ was observed in T cells, NK cells, and NKT cells after the infection (**Figure 4B**). IFNGR is a heterodimeric complex that is comprised of IFNGR1 and IFNGR2. Macrophages were identified as the cluster expressing high levels of IFNGR1 and IFNGR2 (**Figures 4C, D**), then we investigated if macrophages responded to IFN-γ and were regulated by IFN-γ after the infection. There were 474 differentially expressed genes including 213 upregulated genes and 261 downregulated genes that were found in splenic macrophages from wild-type infected mice compared with wild-type uninfected mice (**Figures 4E, G**). To identify the biological function of upregulated genes, GO enrichment analysis was performed and revealed that some of the upregulated genes involved in “respond to IFN-gamma”, “iron ion homeostasis” and “cellular iron ion homeostasis” pathways (**Supplementary Figures S6A-C**). All the upregulated genes involved in “iron ion homeostasis” or “cellular iron ion homeostasis” pathways were labeled (**Figure 4E**), and the heatmap showed that macrophages from wild-type infected mice expressed higher levels of these genes (**Figure 4F**). After the infection, serum iron, transferrin saturation (TS), and total iron binding capacity (TIBC) were increased, however, there was no difference between wild-type and *TLR7* ^-/-^ infected mice (**Supplementary Figures S6D-F**). Hepcidin, a negative regulator of iron export by suppressing ferroportin, and erythroferrone, a positive regulator of iron export by inhibiting hepcidin synthesis, are involved in the iron metabolism but serum erythroferrone and hepcidin were not altered after the infection (**Supplementary Figures S6G, H**). Notably, after the infection, iron was accumulated and stored in the spleen, especially in wild-type infected mice (**Figures 5A, B**), which may be a major source of iron for splenic erythropoiesis in our model. Besides, Fe^2+^ content of macrophages or erythroid cells was detected with FerroOrange. After the infection, the iron content of newly produced splenic ProE was not changed (**Figure 5C**) but the iron content of the CD45^-^ Ter119^+^ population (including EryA, EryB, and EryC) was increased (**Figure 5D**). And the iron content of the CD45^-^ Ter119^+^ population was more abundant in wild-type infected mice than in *TLR7* ^-/-^ infected mice (**Figure 5D**). Meanwhile, the accumulation of iron in red pulp macrophage (RPM) or splenic macrophage was more significantly increased in wild-type infected mice than in *TLR7* ^-/-^ infected mice (**Figures 5E, F**). These findings indicated a hint that upregulated iron metabolism in splenic macrophages is a possible link between IFN-γ and extramedullary splenic erythropoiesis.

### IFN-γ promotes RAW264.7 to phagocytose iRBCs *in vitro*

**Figure 6:**
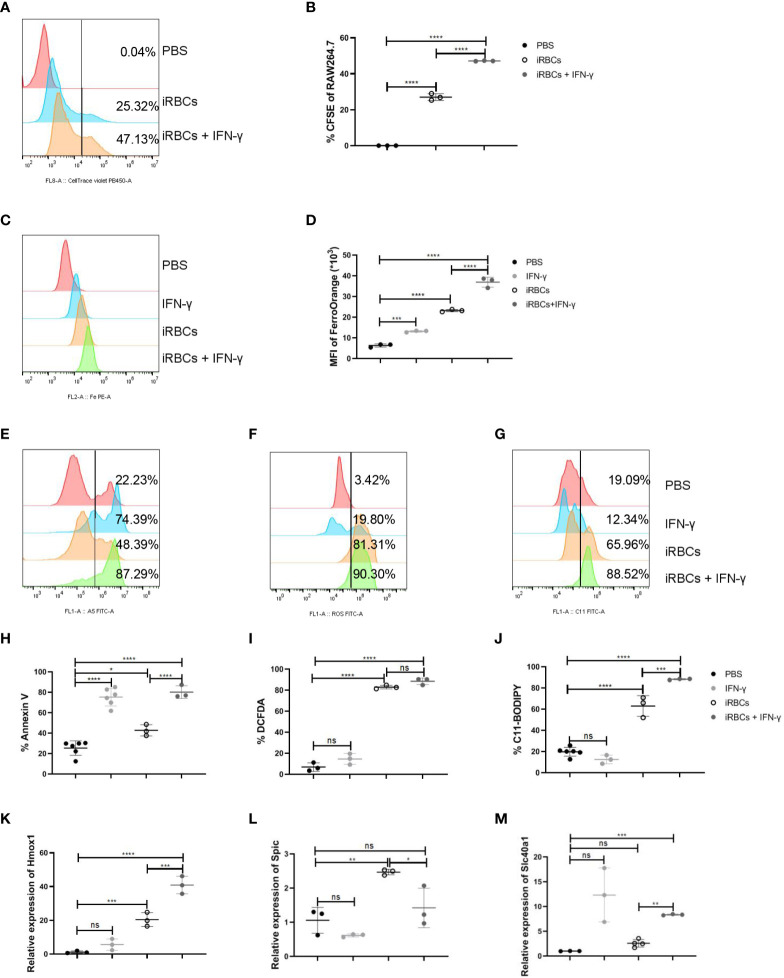
IFN-γ enhances the phagocytosis of iRBCs by RAW264.7 and upregulates the regulators associated with iron metabolism *in vitro*. RAW264.7 cells were cocultured with labeled iRBCs at the ratio of 1:3 with or without IFN-γ treatment for 5 h. **(A)** The representative histogram shows the percentage of CFSE in RAW264.7 cells and **(B)** the percentage of CFSE in RAW264.7 were analyzed. RAW264.7 cells were stimulated with PBS as a control, or with IFN-γ (200 ng/ml), iRBCs (iRBCs: cells = 3:1), IFN-γ plus iRBCs for 20 h respectively. After that, **(C, D)** the mean fluorescence intensity of FerroOrange was detected and analyzed; **(E, H)** the percentage of Annexin V^+^ RAW264.7 cells was measured and analyzed; **(F, I)** ROS in RAW264.7 cells was measured and analyzed; **(G, J)** Lipid peroxidation in RAW264.7 cells was measured and analyzed; **(K-M)** Relative *Hmox1*, *Spic* and *Slc40a1* mRNA levels in RAW264.7 cells were measured by qPCR respectively. **(B)** n=3 samples per group; **(D, H-J)** n=3-6 samples per group; **(K-M)** n=3 samples per group. Data shown as mean ± SEM are representative of three independent experiments; *P < 0.05, **P < 0.01,***P < 0.001, ****P < 0.0001, ns, not significant, *P >*0.05; ANOVA with Sidak multiple comparisons test.

RAW264.7 were cocultured with iRBCs to illustrate the regulation of macrophages by IFN-γ. Before RAW264.7 were cocultured with CFSE-labelled uRBCs or iRBCs, the efficiency of labeling in uRBCs or iRBCs was assessed. All uRBCs and iRBCs were labeled with CFSE successfully (**Supplementary Figure S7A**) and iRBCs were more sensitive to being phagocytosed by RAW264.7 than uRBCs *in vitro* (**Supplementary Figures S7B, C**). When cocultured with iRBCs, IFN-γ could significantly enhance the phagocytosis of iRBCs by RAW264.7 (**Figures 6A, B**). After phagocytosis, iRBCs are digested by ROS and hydrolytic enzymes, and then heme is released from hemoglobin (39). Subsequently, heme oxygenase-1 (HO-1) cleaves heme to produce equimolar amounts of iron, carbon monoxide, and biliverdin (39). Therefore, Fe^2+^ was increased in RAW264.7 after phagocytosis of iRBCs, which could be more significant with the stimulation of IFN-γ (**Figures 6C, D**). Ferroptosis, a type of regulated necrosis dependent on iron, could be indicated by the levels of annexin V, ROS, and lipid peroxidation, and ferroptosis of RPM was induced by increased phagocytosis of RBCs in the murine model of transfusion (40). In our model, macrophages were responsible to phagocytose iRBCs. IRBCs, but not uRBCs, could induce a high level of annexin V, ROS, and lipid peroxidation in RAW264.7 *in vitro*, indicating the ferroptosis of RAW264.7 (**Supplementary Figures S7D-I**). It’s likely that ferroptosis of RAW264.7 was induced by phagocytosis of iRBCs and the accumulation of iron. As IFN-γ augmented the phagocytosis of iRBCs by RAW264.7, higher levels of annexin V and lipid peroxidation were observed in RAW264.7 (**Figures 6E–J**). However, the level of ROS in RAW264.7 was not increased by enhanced phagocytosis of iRBCs (**Figures 6F, I**). HO-1 that cleaves heme to release iron, Spic that is a transcription factor to promote Slc40a1 expression, and Slc40a1 that is the sole mammalian non-heme iron exporter, were associated with iron metabolism and upregulated in macrophages after the infection (**Figures 4E, F**). The relative mRNA levels of *HO-1*, *Spic*, and *Slc40a1* were measured to highlight the iron metabolism in RAW264.7 after phagocytosis. Treated with IFN-γ, relative mRNA levels of *HO-1* and *Slc40a1* in RAW264.7 that were cocultured with iRBCs were increased, and the relative mRNA level of *Spic* was decreased because IFN-γ can suppress the expression of *Spic* (**Figures 6K-M**). These findings indicated that IFN-γ enhances phagocytosis of iRBCs by RAW264.7 *in vitro*, which may facilitate iron metabolism subsequently.

### Neutralization of IFN-γ modestly impedes the extramedullary splenic erythropoiesis in *P. yoelii* NSM-infected mice

**Figure 7:**
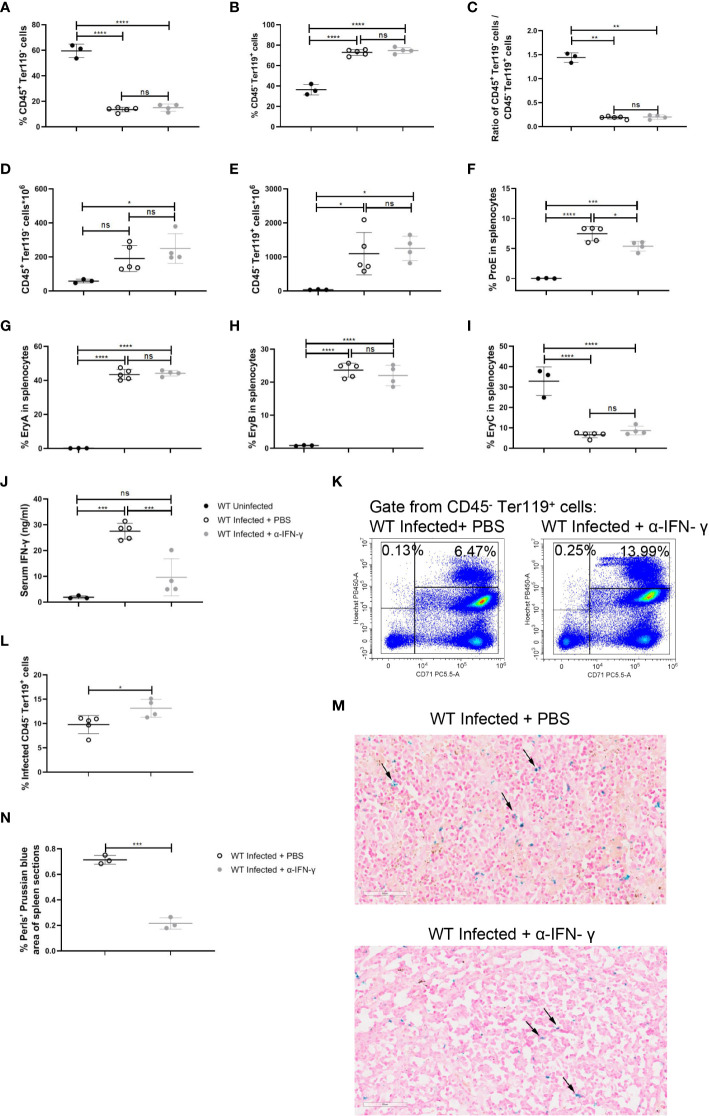
Neutralization of IFN-γ has a modest effect on splenic extramedullary erythropoiesis in *P. yoelii NSM*-infected C57BL/6 mice. **(A)** Percentage and **(D)** the absolute number of CD45^+^ Ter119^-^ cells in spleens from wild-type uninfected, infected, and infected and α-IFN-γ-treated mice were analyzed at 13 dpi. **(B)** Percentage and **(E)** the absolute number of CD45^-^ Ter119^+^ cells in spleens from wild-type uninfected, infected, and infected and α-IFN-γ-treated mice were analyzed at 13 dpi. **(C)** The ratio of the percentage of CD45^+^ Ter119^-^ cells over the percentage of CD45^-^ Ter119^+^ cells of wild-type uninfected, infected, and infected and α-IFN-γ-treated mice were analyzed at 13 dpi. **(F-I)** Percentages of ProE, EryA, EryB, and EryC in splenocytes from wild-type uninfected, infected, and infected and α-IFN-γ-treated mice were analyzed at 13 dpi. **(J)** IFN-γ levels of serum from wild-type uninfected, infected, and infected and α-IFN-γ-treated mice were detected by ELISA at 13dpi. **(K)** Representative pseudocolor plots of infected CD45^-^ Ter119^+^ cells including CD71^-^ Hoechst^low^ and CD71^+^ Hoechst^hi^ cells. **(L)** The percentage of infected CD45^-^ Ter119^+^ cells in spleens from wild-type infected, and infected and α-IFN-γ-treated mice were analyzed at 13dpi. **(M)** Perls’ Prussian blue staining in sections of spleens from wild-type infected, and infected and α-IFN-γ-treated mice. **(N)** The percentage of Perls’ Prussian blue area in spleen sections from wild-type infected, and infected and α-IFN-γ-treated mice were calculated and analyzed at 13dpi. **(A- I)** n=3-5 mice per group; **(J)** n=3-5 mice per group; **(L)** n=3-5 mice per group; **(N)** n=3 mice per group. Data shown as mean ± SEM are representative of three independent experiments; *P < 0.05, **P < 0.01,***P < 0.001, ****P < 0.0001, ns, not significant, *P >*0.05; ANOVA with Sidak multiple comparisons test or unpaired t-test.

To emphasize the impact of IFN-γ on the splenic extramedullary erythropoiesis in wild-type infected mice, neutralization of IFN-γ *in vivo* was performed after the infection. Neutralization of IFN-γ did not affect the percentage of splenic CD45^+^ Ter119^-^ cells or splenic CD45^-^ Ter119^+^ cells in wild-type infected mice (**Figures 7A-C**). And neutralization of IFN-γ did not alter the absolute number of splenic CD45^+^ Ter119^-^ cells or CD45^-^ Ter119^+^ cells (**Figures 7D, E**). Contrary to the treatment of R848, neutralization of IFN-γ reduced the percentage of ProE in wild-type infected mice (**Figure 7F**), while the percentages of EryA, EryB, and EryC were not altered (**Figures 7G-I**). Previous reports suggested that IFN-γ could enhance the phagocytosis of RBCs by macrophages (29, 30). We also confirmed that IFN-γ could enhance phagocytosis of iRBCs by RAW264.7 *in vitro* (**Figures 6A, B**). Associatively, after neutralization of IFN-γ, serum IFN-γ was reduced significantly (**Figure 7J**), and a slight increase in the percentage of infected CD45^-^ Ter119^+^ cells in the spleen was observed (**Figures 7K, L**). Given that phagocytosis of RBCs is of great importance in iron recycling and iron homeostasis (41), Perls’ Prussian blue staining was performed to assess the accumulation of iron in the spleen. The result showed that the accumulation of iron in the spleen decreased after the neutralization of IFN-γ (**Figures 7M, N**). All of these suggested that the neutralization of IFN-γ significantly reduced the iron accumulation in the spleen and slightly hampered the extramedullary splenic erythropoiesis.

### TLR7 regulates the phagocytosis of iRBCs in macrophages and RAW264.7

Splenic macrophages expanded dramatically after the infection and displayed a higher level of TLR7 expression (**Supplementary Figures S8A-D**), indicating that macrophages responded to TLR7 ligand as well. Given the effect of Myd88 on erythrophagocytosis (14), TLR7 signaling may regulate the functionality of macrophages. Compared with splenic macrophages from *TLR7* ^-/-^ mice, splenic macrophages from wild-type mice phagocytosed iRBCs more powerfully *in vitro* (**Supplementary Figures S8E, F**). Additionally, R848 could significantly enhance the phagocytosis of iRBCs by RAW264.7 *in vitro* (**Supplementary Figures S9A, B**). However, stimulation with R848 could not alter the level of annexin V, ROS, and lipid peroxidation in RAW264.7 that cocultured with iRBCs (**Supplementary Figures S9C-H**). And stimulation with R848 did not alter *HO-1*, *Spic*, and *Slc40a1* expression in RAW264.7 that cocultured with iRBCs (**Supplementary Figures S9I-K**). These suggested that TLR7 signaling could also promote the phagocytosis of iRBCs in macrophages and RAW264.7.

## Discussion

Extramedullary splenic erythropoiesis is a critical mechanism for replenishing erythrocytes that are reduced by the limited output of erythropoiesis in the bone marrow and the great loss of erythrocytes (42). It’s reported that inflammation suppressed the steady state erythropoiesis by shifting hematopoiesis toward the production of myeloid cells to control the infection (43). As the inflammatory response is initiated and numerous erythrocytes are destructed, splenic erythropoiesis is observed in malaria (44). In our model, the percentage of erythroid progenitor cells was increased in bone marrow (**Supplementary Figures S3D-G**). A similar finding was shown by *Auclair et al.* (8). Erythroid progenitors, CFU-E but not BFU-E, were increased in the bone marrow of *P. berghei*-infected mice at 8 dpi. (8). Anemia of malaria was considerably severe, and the number of RBCs was lower than 5×10^12^/L at 16 dpi. (**Figure 1B**), which may strongly mobilize the bone marrow erythropoiesis. Nevertheless, that was still not enough for compensating the great loss of RBCs, leading to anemia in the *P. yoelii* NSM-infected mice. Bone marrow erythropoiesis in malaria seemed to be suppressed partially or not to be suppressed. It’s plausible that bone marrow erythropoiesis in infected mice will be mobilized more robustly when an anti-inflammation intervention is performed. Splenomegaly (**Figures 1C, D**) and the increased number of erythroid progenitor cells in the spleen (**Figures 1G**) were observed in *P. yoelii* NSM-infected mice, indicating that extramedullary splenic erythropoiesis was induced by malaria. Moreover, the percentage of splenic immune cells was decreased but the percentage of splenic erythroid cells was increased following the infection of *P. yoelii* NSM (**Figures 1F, G**), and most splenic erythroid cells expressed CD71 (**Figure 1J**), suggesting that the expansion of erythroid progenitor cells contributed mostly to splenomegaly in the *P. yoelii* NSM-infected mice. Besides, the absolute number of splenic immune cells was increased (**Figure 1H**), which also likely correlated with splenomegaly. The expression of TLR7 in splenic CD45^+^ cells was increased after the infection (**Figure 2B**). Single-cell RNA sequencing of splenic immune cells revealed that among all TLRs, *TLR7* mRNA was expressed relatively highly at 12 dpi. and DCs and macrophages were the primary populations expressing TLR7, although the expression level was reduced (**Supplemental Table S1**). The transcription and translation were not parallel. The splenic erythropoiesis in *TLR7* ^-/-^ infected mice was suppressed compared to the wild-type infected mice (**Figure 2G**), which implied that TLR7 affected the development of erythroid progenitor cells.

After *Plasmodium* infection, ssRNA of *Plasmodium* could trigger TLR7 signaling and induce Th1 response and IFN-γ response (25, 38). In parallel with previous reports, IFN-γ production in the spleen was dramatically promoted in wild-type infected mice, and T cell was the primary population producing IFN-γ (**Figure 3**). However, IFN-γ production was suppressed in *TLR7* ^-/-^ infected mice (**Figure 3**). IFN-γ could augment the expression of MHC I and MHC II to promote antigen-presentation of APCs, such as DCs, macrophages, and B cells (45). Here, in our murine malaria model, responding to IFN-γ, splenic macrophages upregulated not only the phagocytosis of RBCs but also the subsequent iron metabolism (**Supplementary Figure S6A**). It was also suggested by RAW264.7 *in vitro*. Stimulated with IFN-γ, phagocytosis of iRBCs was more robust and the genes associated with iron metabolism were upregulated (**Figure 6**). It was reported that IFN-γ could reduce the life span of erythrocytes and inhibit steady-state erythropoiesis, inducing anemia in the inflammatory setting (16). A different effect of IFN-γ on erythropoiesis was shown by our work. Given the effect of IFN-γ on macrophages, the neutralization of IFN-γ modestly reduced splenic erythropoiesis in infected mice (**Figure 7**). IFN-γ-upregulated phagocytosis of iRBCs and iron metabolism may be potential contributors to malaria-induced splenic erythropoiesis. Being opposite to the effect of TLRs, IFN-γ could inhibit the expression of *Spic* in macrophages (46). TLRs signaling is strongly activated after the infection and may counteract the effect of IFN-γ on the expression of *Spic* in our model. Additionally, TLR7 directly promoted the phagocytosis of iRBCs in splenic macrophages and RAW264.7 *in vitro* (**Supplementary Figures S8F**, **S9B**). As a previous study showed, Myd88 could promote macrophage phagocytosis by blocking the SIRPα-CD47 axis (14). However, enhanced phagocytosis of iRBCs by R848 was unable to further upregulate the genes associated with iron metabolism in RAW264.7 (**Supplementary Figures S9I-K**), which could be attributed to a less powerful impact of TLR7 on phagocytosis.

The iron that is supplied from diet and iron recycling in the spleen is essential for heme synthesis in developing erythroid progenitor cells (39, 47). Given the *Plasmodium*-infected cells that were primarily eliminated in the spleen (35), large amounts of iron could be observed in the spleen after the infection. Commonly, the accumulation of iron in the spleen was used as an indicator of iron sequestration (24). As the expression of *slc40a1* in splenic macrophages, serum iron, TIBC, and TS% increased after the infection, accumulation of iron in the spleen is more appropriate to use as an indicator of iron overload in the spleen for our model. Iron accumulated in the spleen, and the iron content of erythroid cells and splenic macrophages were less in *TLR7* ^-/-^ infected mice than in wild-type infected mice (**Figure 5**), suggesting that TLR7 is associated with iron metabolism in malaria. A previous study found that TLRs can upregulate *Spic* expression in macrophages through NF-κB and STAT signaling to control iron metabolism (46). Here we discovered another potential mechanism by which TLR7 regulates the iron metabolism of macrophages. The production of IFN-γ in the infected mice was promoted by TLR7 (**Figure 3**). *In vitro*, IFN-γ could enhance the phagocytosis of iRBCs by RAW264.7, increased the iron content, and upregulated the genes associated with iron metabolism (**Figure 6**). *In vivo*, the infected mice that were treated with anti-IFN-γ showed an increased percentage of infected erythrocytes (**Figure 7L**), lower iron accumulation in the spleen (**Figure 7N**), and a modest decrease in the percentage of erythroid progenitor cells in the spleen (**Figure 7F**). Therefore, promoting the production of IFN-γ to enhance the phagocytosis of infected erythrocytes by macrophages is a potential mechanism of iron metabolism regulation by TLR7. Because there were differences in genome and transcriptome between RAW264.7 and splenic macrophages, *in vivo* evidence is needed to further confirm the effect of IFN-γ on macrophage iron metabolism. Phagocytosis indicated by GFP-expressing or fluorescence-labeled *Plasmodium* is appropriate to set for splenic macrophages. Moreover, blocking the slc40a1 in macrophages after the infection may clarify that iron recycling is the mechanism by which splenic macrophages regulate malaria-induced splenic erythropoiesis, though *Zhang et al.* have reported that iron exported by macrophages is indispensable for erythropoiesis (24).

Notably, knockout of TLR7, R848 treatment, and the neutralization of IFN-γ affect the relatively early stage of erythroid progenitor cells with high expression of CD71, especially the ProE and EryA. CD71, a transferrin receptor responsible for the binding and endocytosis of transferrin, is expressed on the erythroid progenitor cells, and the expression level is correlated with the demand of iron which is an element for heme synthesis (48). In certain stages, erythroid progenitor cells express a higher level of CD71 for increasing heme synthesis (48). These also highlighted that TLR7 regulated the malaria-induced splenic erythropoiesis with iron. Other TLRs possibly were involved in the malaria-induced splenic erythropoiesis as TLRs induce the expression of Spic through NF-κB signaling to promote iron metabolism (14, 46). For example, TLR4 and TLR9 could be activated by *Plasmodium* in rodent malaria (49, 50).

Additionally, other factors may be important in malaria-induced erythropoiesis. Inflammatory cytokines are induced during the infection of *Plasmodium*. The production of TNF-α and IL-1β was at the highest level during the early stage of *P. yoelii* infection (49). As TNF-α and IL-1β could support splenic erythropoiesis in the inflammatory setting (14), they might also play a role in malaria-induced splenic erythropoiesis at the early stage of *P. yoelii* infection. Monocytes were recruited to the spleen by CCL2 chemokine and differentiated to RPMs, and BMP4 expressed by RPMs in spleen red pulp provided a key signal for splenic erythroid progenitors expansion (10, 11, 14, 51). BMP4 induction is that heme was accumulated in macrophages after erythrophagocytosis and promoted the degradation of BACH1 that inhibits Spic expression, therefore, Spic was induced to facilitate GDF15 and BMP4 expression (14, 52). RPMs may support malaria-induced erythropoiesis through this mechanism as well.

Similarly, the human spleen is considered as a site for stress erythropoiesis in the case of inflammation (43), prompting us to investigate the role of splenic erythropoiesis in human malaria. Our present study that demonstrates that TLR7 modulates splenic erythropoiesis in malaria gives an insight into the relationship between phagocytosis of iRBCs and splenic erythropoiesis and suggests that TLR7 plays multiple roles in malaria which is a potential target of therapies in malaria.

## Data availability statement

The datasets presented in this study can be found in online repositories. The names of the repository/repositories and accession number(s) can be found below: https://www.ncbi.nlm.nih.gov/, PRJNA889506 https://www.ncbi.nlm.nih.gov/, PRJNA889507.

## Ethics statement

The animal study was reviewed and approved by the institutional animal care and use committee of Guangzhou Medical University.

## Author contributions

JL, LL, and JX were responsible to complete the animal experiments and analyze the results. JL, XP, XY, and JH designed the research. DC, CF, FM, YG, ZT, GL, WX, ST, SZ, HW, HX, XP, XY, and JH guided the research. The final manuscript was written by JL and JH, and other authors reviewed the manuscript. All authors contributed to the article and approved the submitted version.

## Supplementary material

Supplementary Figure 1 The knockout of *TLR7* causes an aggravation of malaria-induced anemia. **(A)** Parasitemia of wild-type and *TLR7* ^-/-^ infected mice was measured at 16 dpi. The hematologic parameters, including **(B)** the number of RBCs, **(C)** hemoglobin (Hgb), **(D)** hematocrit (HCT), **(E)** mean corpuscular volume (MCV), **(F)** mean corpuscular hemoglobin (MCH), **(G)** mean corpuscular hemoglobin concentration (MCHC), and **(H)** red blood cell distribution width (RDW), of wild-type uninfected and infected, *TLR7* ^-/-^ uninfected and infected mice were detected at 16 dpi. **(A)** n=11-13 mice per group; **(B-H)** n=3-11 mice per group; Data shown as mean ± SEM are representative of three independent experiments; *P < 0.05, **P < 0.01,***P < 0.001, ****P < 0.0001, ns: not significant, P >0.05; ANOVA with Sidak multiple comparisons test or unpaired t-test.

Supplementary Figure 2 Stages of erythroid progenitor cells. Representative pseudocolor plots from wild-type uninfected and infected, *TLR7* ^-/-^ uninfected and infected mice show the different stages of erythroid progenitor cells including ProE, EryA, EryB, and EryC.

Supplementary Figure 3 TLR7 does not modulate intramedullary erythropoiesis in the bone marrow of *P. yoelii* NSM-infected C57BL/6 mice. **(A)** The percentage of CD45^+^ Ter119^-^ cells in bone marrow from wild-type uninfected and infected, *TLR7* ^-/-^ uninfected and infected mice were analyzed at 16 dpi. **(B)** The percentage of CD45^-^ Ter119^+^ cells in the bone marrow from wild-type uninfected and infected, *TLR7* ^-/-^ uninfected and infected mice were analyzed at 16 dpi. **(C)** The ratio of CD45^+^ Ter119^-^ percentage over CD45^-^ Ter119^+^ percentage of wild-type uninfected and infected, *TLR7* ^-/-^ uninfected and infected mice were analyzed at 16 dpi. **(D-G)** Percentages of ProE, EryA, EryB, or EryC in bone marrow cells from wild-type uninfected and infected, *TLR7* ^-/-^ uninfected and infected mice were analyzed at 16 dpi. **(H)** The percentage of Annexin V^+^ CD45^-^ CD71^+^ cells in bone marrow from wild-type uninfected and infected, *TLR7* ^-/-^ uninfected and infected mice were analyzed at 16 dpi. **(I)** Gating strategy for CD45^-^ cells in the bone marrow and the representative histogram shows TLR7 expression level in CD45^-^ cells of wild-type infected mice at 16 dpi. **(A-G)** n=3-11 mice per group; **(H)** n=4-8 mice per group; Data shown as mean ± SEM are representative of three independent experiments; **P* < 0.05, ***P* < 0.01,****P* < 0.001, *****P* < 0.0001, ns: not significant, *P >*0.05; ANOVA with Sidak multiple comparisons test.

Supplementary Figure 4 Treatment of R848 promotes extramedullary splenic erythropoiesis in *P. yoelii* NSM-infected C57BL/6 mice. **(A)** Percentage and **(D)** the absolute number of CD45^+^ Ter119^-^ cells in spleens from wild-type uninfected, infected, and infected and R848-treated mice were analyzed at 12dpi. **(B)** Percentage and **(E)** the absolute number of CD45^-^ Ter119^+^ cells in spleens from wild-type uninfected, infected, and infected and R848-treated mice were analyzed at 12dpi. **(C)** The ratio of the percentage of CD45^+^ Ter119^-^ cells over the percentage of CD45^-^ Ter119^+^ cells of wild-type uninfected, infected, and infected and R848-treated mice were analyzed at 12dpi. **(F-I)** Percentages of ProE, EryA, EryB, or EryC in splenocytes from wild-type uninfected, infected, and infected and R848-treated mice were analyzed at 12dpi. **(A-I)** n=3-12 mice per group. Data shown as mean ± SEM are representative of three independent experiments; *P < 0.05, **P < 0.01,***P < 0.001, ****P < 0.0001, ns: not significant, P >0.05; ANOVA with Sidak or Tamhane T2 multiple comparisons test.

Supplementary Figure 5
*P. yoelii* NSM-infected cells are observed in the spleen, bone marrow, and blood. **(A)** Representative pseudocolor plots of infected CD45^-^ Ter119^+^ cells including CD71^-^ Hoechst^low^ and CD71^+^ Hoechst^hi^ cells in the spleen, bone marrow, and blood of wild-type infected mice. **(B, C)** The percentage of infected CD45^-^ Ter119^+^ cells in the spleen, bone marrow, and blood from wild-type infected mice and *TLR7* ^-/-^ infected mice were analyzed at 16 dpi. respectively. **(B, C)** n=8 mice per group. Data shown as mean ± SEM are representative of three independent experiments; *P < 0.05, **P < 0.01, ns: not significant, P >0.05; RM one-way ANOVA with Tukey multiple comparisons test.

Supplementary Figure 6 Iron metabolism is upregulated after *P. yoelii* NSM infection. **(A-C)** Gene Ontology (GO) analysis of significantly upregulated genes in macrophages from wild-type infected mice compared with wild-type uninfected mice. **(A)** Enrichment of GO terms involved in biological process. **(B)** Enrichment of GO terms involved in the cellular component. **(C)** Enrichment of GO terms involved in molecular function. **(D-F)** Iron metabolism indicators including serum iron, TS, and TIBC were detected in serum from wild-type uninfected and infected, *TLR7* ^-/-^ uninfected and infected mice at 16 dpi. **(G)** Serum erythroferrone levels of wild-type uninfected and infected, *TLR7* ^-/-^ uninfected and infected mice were detected by ELISA at 16 dpi. **(H)** Serum hepcidin levels of wild-type uninfected and infected, *TLR7* ^-/-^ uninfected and infected mice were detected by ELISA at 16 dpi. **(D-F)** n=3-5 mice per group; **(G-H)** n=3-4 mice per group. Data shown as mean ± SEM are representative of three independent experiments; *P < 0.05, ***P < 0.001, ****P < 0.0001, ns: not significant, P >0.05; ANOVA with Sidak multiple comparisons test.

Supplementary Figure 7 RAW264.7 phagocytose iRBCs efficiently *in vitro*. **(A)** The representative histogram indicates that uRBCs and iRBCs were both labeled by CellTrace CFSE. RAW264.7 cells were cocultured with unlabeled uRBCs, labeled uRBCs, unlabeled iRBCs, and labeled iRBCs respectively at the ratio of 1:5 for 5 h. **(B,C)** The percentage of CFSE in RAW264.7 cells was measured and analyzed. **(D, G)** The percentage of Annexin V^+^ RAW264.7 cells was measured and analyzed. **(E, H)** ROS in RAW264.7 cells was measured and analyzed. **(F, I)** Lipid peroxidation in RAW264.7 cells was measured and analyzed. **(C)** n=3 samples per group; **(G-I)** n=3 samples per group. Data shown as mean ± SEM are representative of three independent experiments; *P < 0.05, **P < 0.01,***P < 0.001, ****P < 0.0001, P >0.05; ANOVA with Sidak multiple comparisons test.

Supplementary Figure 8 The knockout of *TLR7* suppresses the phagocytosis of iRBCs by macrophages *in vitro*. **(A)** Representative pseudocolor plots of splenic macrophages. **(B)** Representative histograms of the TLR7 expression in splenic macrophages. **(C)** The percentage of splenic macrophages in wild-type uninfected and infected, *TLR7* ^-/-^ uninfected and infected mice were analyzed at 16 dpi. **(D)** Expression levels of TLR7 in splenic macrophages from wild-type uninfected and infected mice were analyzed at 16 dpi. Splenocytes from wild-type and *TLR7* ^-/-^ uninfected mice were cocultured with labeled uRBCs or labeled iRBCs at the ratio of 1: 1 for 5 h. **(E)** The representative histogram shows the percentage of CFSE in macrophages cocultured with uRBCs or iRBCs, and **(F)** the percentage of CFSE in macrophages was analyzed. **(C)** n=7-13 mice per group; **(D)** n=5-7 mice per group; **(F)** n=3-6 samples per group. Data shown as mean ± SEM are representative of three independent experiments; *P < 0.05, **P < 0.01,***P < 0.001, ****P < 0.0001, ns: not significant, P >0.05; ANOVA with Sidak multiple comparisons test or unpaired t-test.

Supplementary Figure 9 R848 augments the RAW264.7 phagocytosis of iRBCs *in vitro*. RAW264.7 cells were cocultured with labeled iRBCs at the ratio of 1:3 with or without R848 treatment for 5 h. **(A)** The representative histogram shows the percentage of CFSE in RAW264.7 cells, and **(B)** the percentage of CFSE in RAW264.7 was analyzed. RAW264.7 cells were stimulated with PBS as a control, or with R848 (2 µg/ml), iRBCs (iRBCs: cells = 3: 1), and R848 plus iRBCs for 20 h respectively. After that, **(C, F)** The percentage of Annexin V^+^ RAW264.7 cells was measured and analyzed; **(D, G)** ROS in RAW264.7 cells was measured and analyzed; **(E, H)** lipid peroxidation in RAW264.7 cells was measured and analyzed; **(I-K)** Relative *Hmox1*, *Spic* and *Slc40a1* mRNA levels in RAW264.7 cells were measured by qPCR respectively. **(B)** n=3 samples per group; **(F-H)** n=3-6 samples per group; **(I-K)** n=3-6 samples per group. Data shown as mean ± SEM are representative of three independent experiments; *P < 0.05, **P < 0.01, ****P < 0.0001, ns: not significant, P >0.05; ANOVA with Sidak multiple comparisons test.

Supplementary Table 1 Mean of Log2 Exp of TLRs. A matrix from Single-cell RNA sequencing showed the mean of Log2 Expression of different TLRs in various cells from wild-type mice before and after the infection.
