# Supplementary figures and images for: TLR7 modulates extramedullary splenic erythropoiesis in P. yoelii NSM-infected mice through the regulation of iron metabolism of macrophages with IFN-γ
Source: Front Immunol. 2023 Apr 27;14:1123074. doi: 10.3389/fimmu.2023.1123074 (PMC10174296; doi:10.3389/fimmu.2023.1123074)

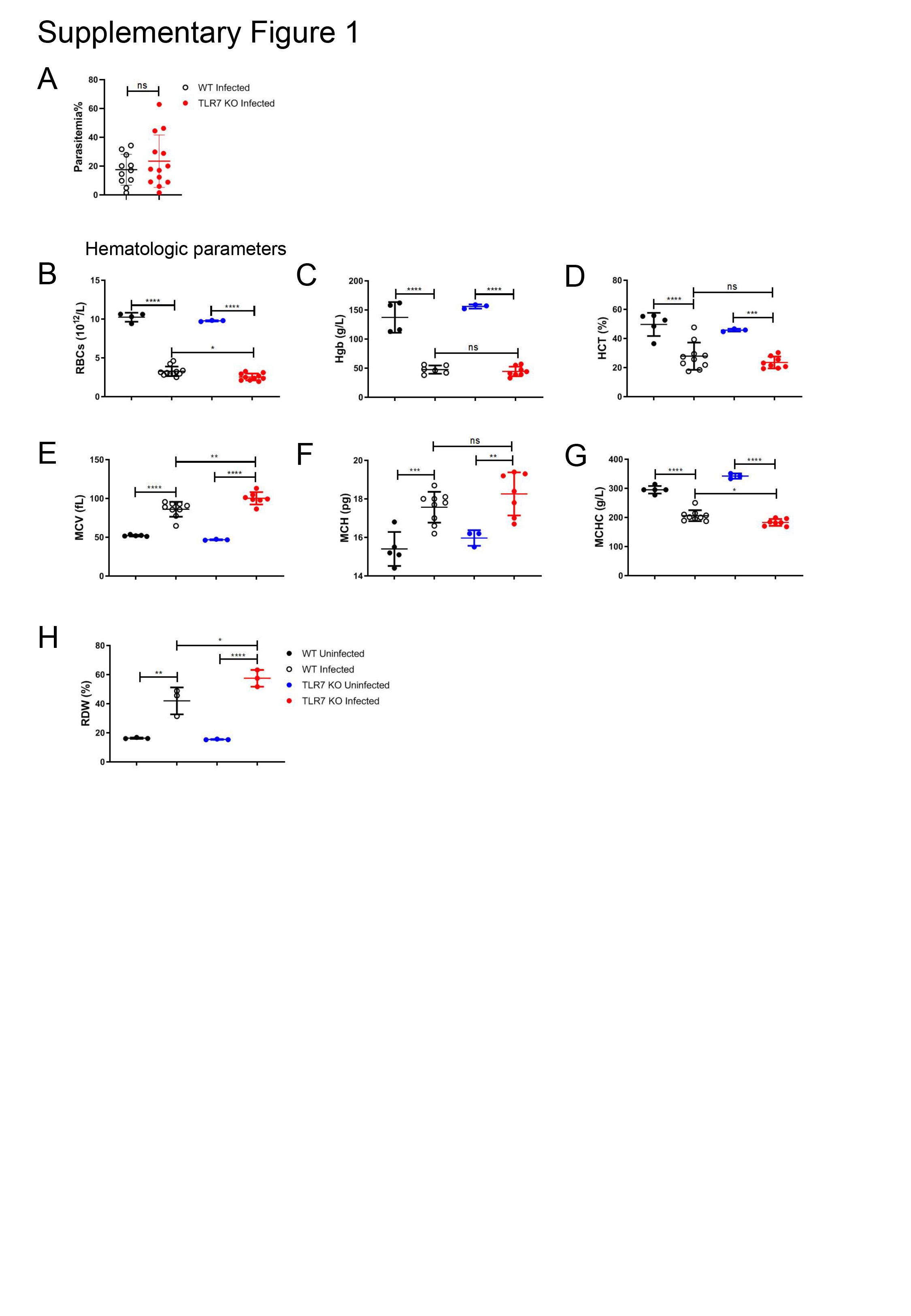

Supplement: Supplementary Figure 1 — The knockout of TLR7 causes an aggravation of malaria-induced anemia. (A) Parasitemia of wild-type and TLR7 -/- infected mice was measured at 16 dpi. The hematologic parameters, including (B) the number of RBCs, (C) hemoglobin (Hgb), (D) hematocrit (HCT), (E) mean corpuscular volume (MCV), (F) mean corpuscular hemoglobin (MCH), (G) mean corpuscular hemoglobin concentration (MCHC), and (H) red blood cell distribution width (RDW), of wild-type uninfected and infected, TLR7 -/- uninfected and infected mice were detected at 16 dpi. (A) n=11-13 mice per group; (B-H) n=3-11 mice per group; Data shown as mean ± SEM are representative of three independent experiments; *P < 0.05, **P < 0.01,***P < 0.001, ****P < 0.0001, ns: not significant, P >0.05; ANOVA with Sidak multiple comparisons test or unpaired t-test. [file Image_1.tif]

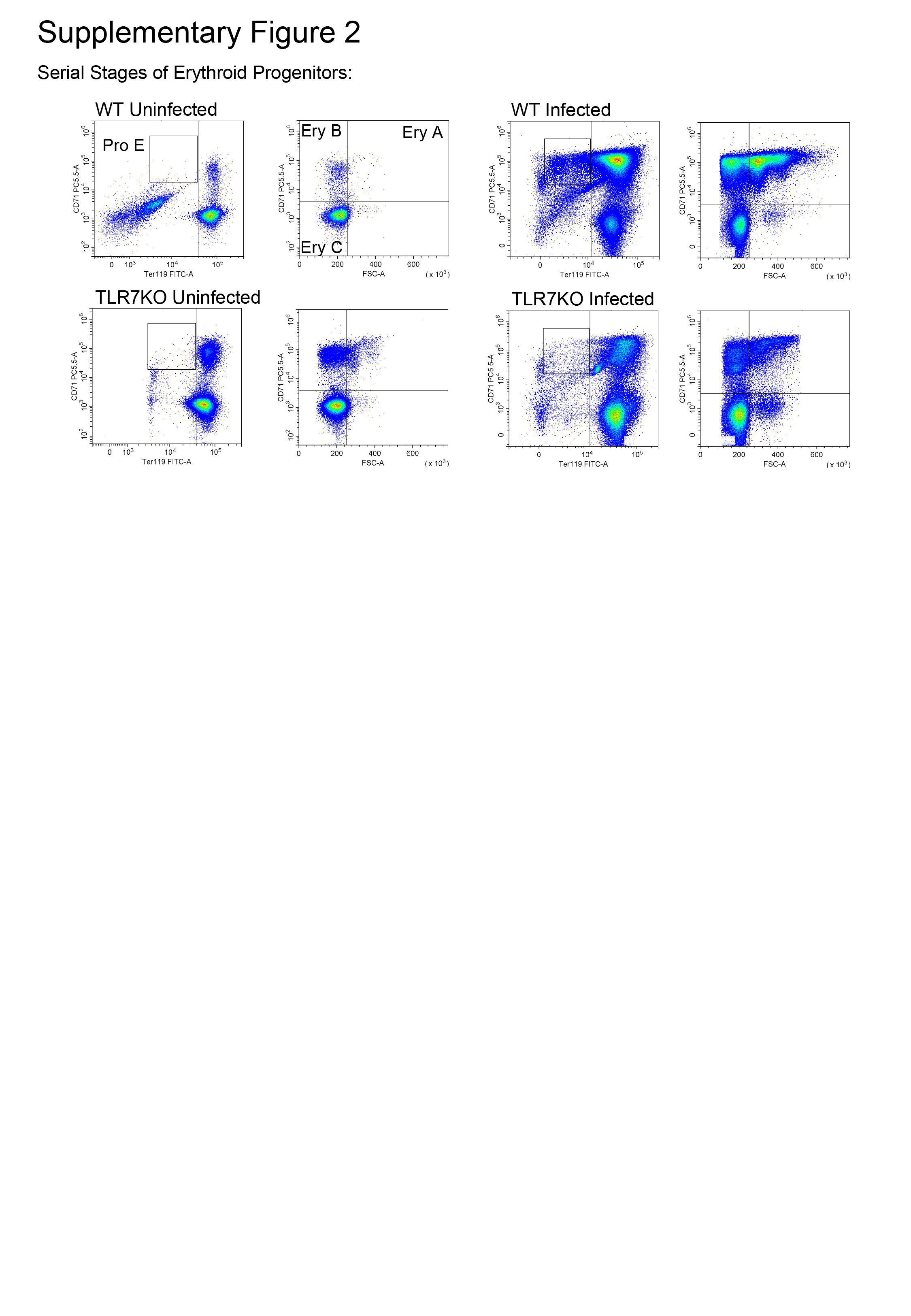

Supplement: Supplementary Figure 2 — Stages of erythroid progenitor cells. Representative pseudocolor plots from wild-type uninfected and infected, TLR7 -/- uninfected and infected mice show the different stages of erythroid progenitor cells including ProE, EryA, EryB, and EryC. [file Image_2.tif]

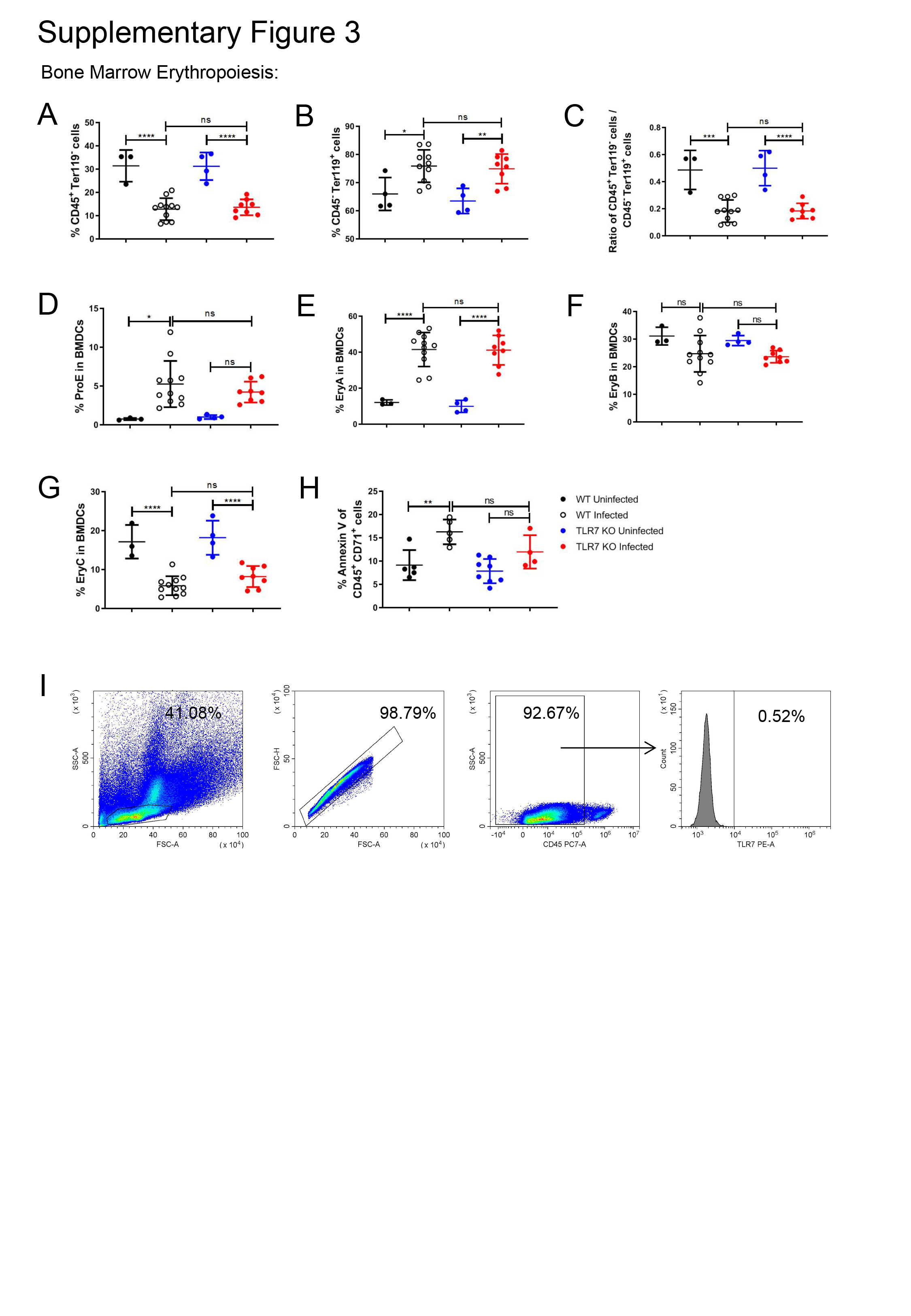

Supplement: Supplementary Figure 3 — TLR7 does not modulate intramedullary erythropoiesis in the bone marrow of P. yoelii NSM-infected C57BL/6 mice. (A) The percentage of CD45+ Ter119- cells in bone marrow from wild-type uninfected and infected, TLR7 -/- uninfected and infected mice were analyzed at 16 dpi. (B) The percentage of CD45- Ter119+ cells in the bone marrow from wild-type uninfected and infected, TLR7 -/- uninfected and infected mice were analyzed at 16 dpi. (C) The ratio of CD45+ Ter119- percentage over CD45- Ter119+ percentage of wild-type uninfected and infected, TLR7 -/- uninfected and infected mice were analyzed at 16 dpi. (D-G) Percentages of ProE, EryA, EryB, or EryC in bone marrow cells from wild-type uninfected and infected, TLR7 -/- uninfected and infected mice were analyzed at 16 dpi. (H) The percentage of Annexin V+ CD45- CD71+ cells in bone marrow from wild-type uninfected and infected, TLR7 -/- uninfected and infected mice were analyzed at 16 dpi. (I) Gating strategy for CD45- cells in the bone marrow and the representative histogram shows TLR7 expression level in CD45- cells of wild-type infected mice at 16 dpi. (A-G) n=3-11 mice per group; (H) n=4-8 mice per group; Data shown as mean ± SEM are representative of three independent experiments; *P < 0.05, **P < 0.01,***P < 0.001, ****P < 0.0001, ns: not significant, P >0.05; ANOVA with Sidak multiple comparisons test. [file Image_3.tif]

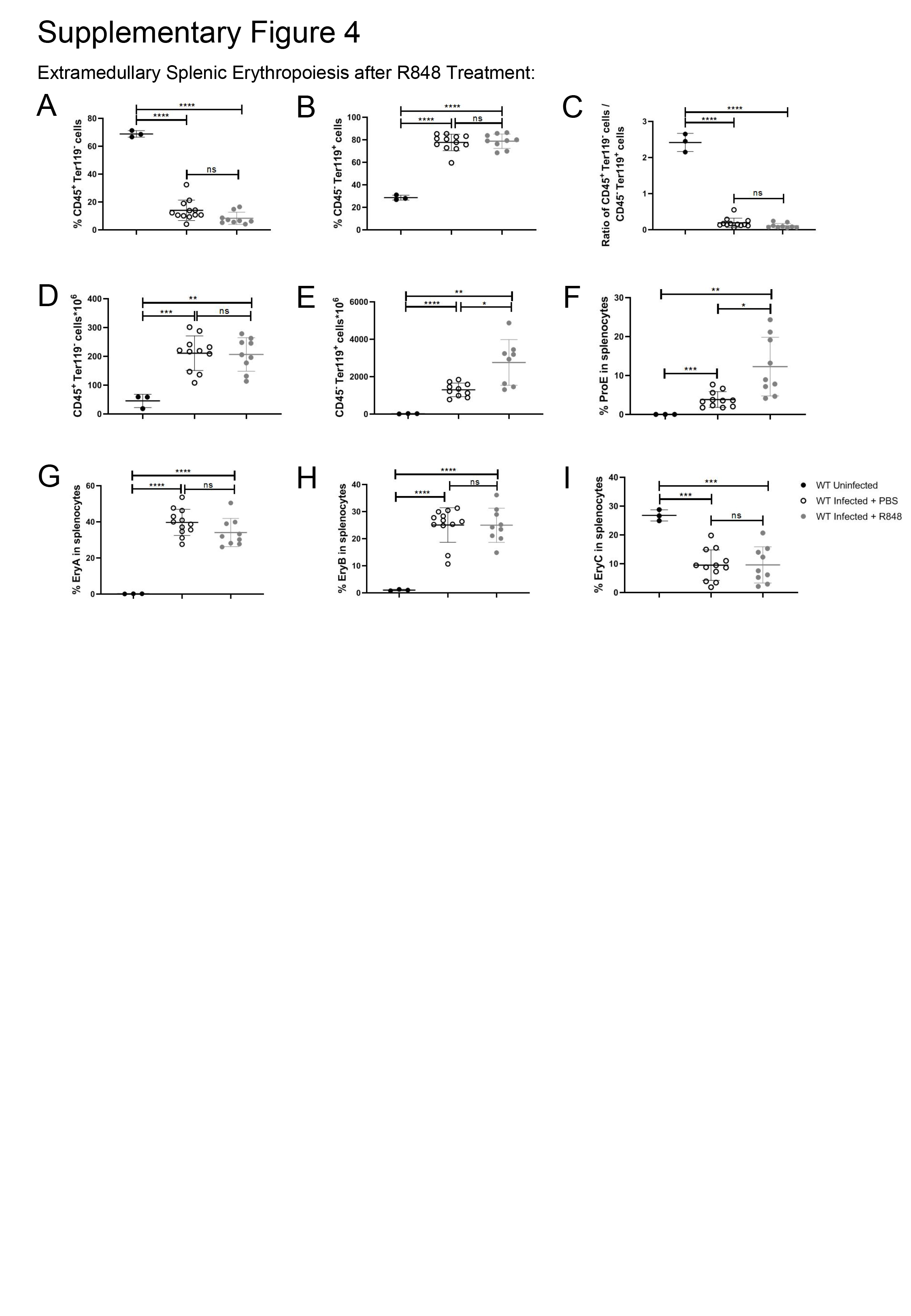

Supplement: Supplementary Figure 4 — Treatment of R848 promotes extramedullary splenic erythropoiesis in P. yoelii NSM-infected C57BL/6 mice. (A) Percentage and (D) the absolute number of CD45+ Ter119- cells in spleens from wild-type uninfected, infected, and infected and R848-treated mice were analyzed at 12dpi. (B) Percentage and (E) the absolute number of CD45- Ter119+ cells in spleens from wild-type uninfected, infected, and infected and R848-treated mice were analyzed at 12dpi. (C) The ratio of the percentage of CD45+ Ter119- cells over the percentage of CD45- Ter119+ cells of wild-type uninfected, infected, and infected and R848-treated mice were analyzed at 12dpi. (F-I) Percentages of ProE, EryA, EryB, or EryC in splenocytes from wild-type uninfected, infected, and infected and R848-treated mice were analyzed at 12dpi. (A-I) n=3-12 mice per group. Data shown as mean ± SEM are representative of three independent experiments; *P < 0.05, **P < 0.01,***P < 0.001, ****P < 0.0001, ns: not significant, P >0.05; ANOVA with Sidak or Tamhane T2 multiple comparisons test. [file Image_4.tif]

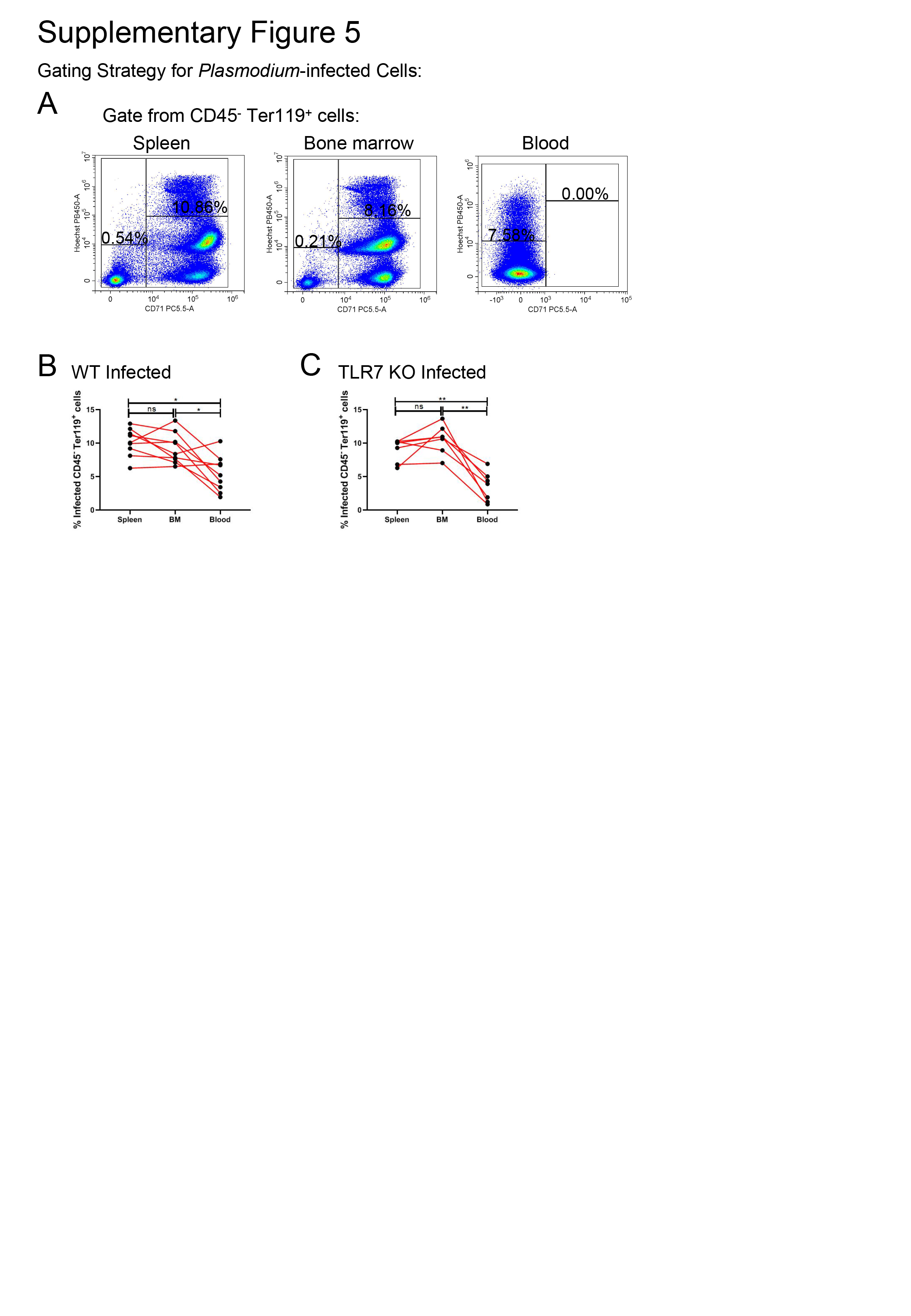

Supplement: Supplementary Figure 5 — P. yoelii NSM-infected cells are observed in the spleen, bone marrow, and blood. (A) Representative pseudocolor plots of infected CD45- Ter119+ cells including CD71- Hoechstlow and CD71+ Hoechsthi cells in the spleen, bone marrow, and blood of wild-type infected mice. (B, C) The percentage of infected CD45- Ter119+ cells in the spleen, bone marrow, and blood from wild-type infected mice and TLR7 -/- infected mice were analyzed at 16 dpi. respectively. (B, C) n=8 mice per group. Data shown as mean ± SEM are representative of three independent experiments; *P < 0.05, **P < 0.01, ns: not significant, P >0.05; RM one-way ANOVA with Tukey multiple comparisons test. [file Image_5.tif]

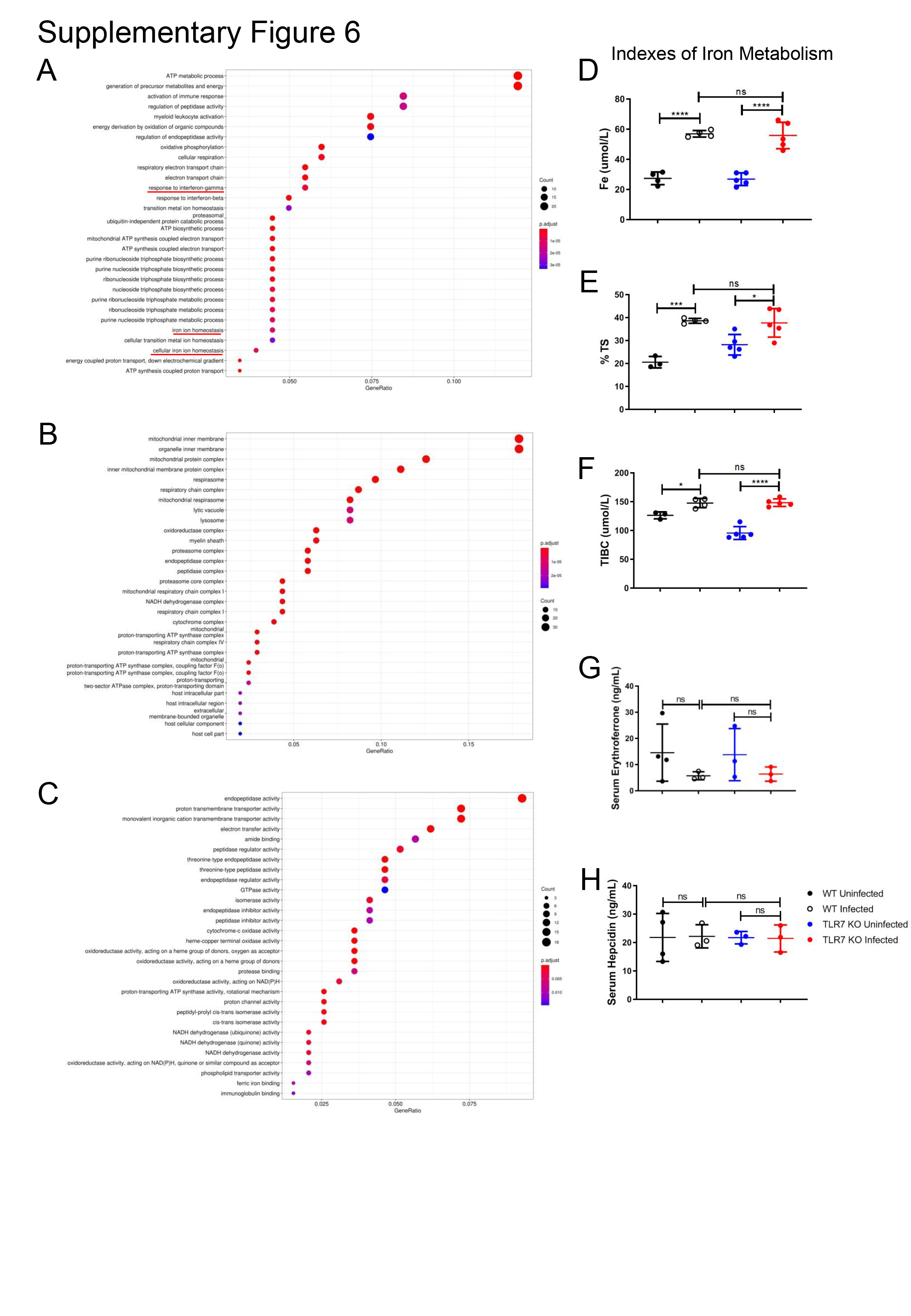

Supplement: Supplementary Figure 6 — Iron metabolism is upregulated after P. yoelii NSM infection. (A-C) Gene Ontology (GO) analysis of significantly upregulated genes in macrophages from wild-type infected mice compared with wild-type uninfected mice. (A) Enrichment of GO terms involved in biological process. (B) Enrichment of GO terms involved in the cellular component. (C) Enrichment of GO terms involved in molecular function. (D-F) Iron metabolism indicators including serum iron, TS, and TIBC were detected in serum from wild-type uninfected and infected, TLR7 -/- uninfected and infected mice at 16 dpi. (G) Serum erythroferrone levels of wild-type uninfected and infected, TLR7 -/- uninfected and infected mice were detected by ELISA at 16 dpi. (H) Serum hepcidin levels of wild-type uninfected and infected, TLR7 -/- uninfected and infected mice were detected by ELISA at 16 dpi. (D-F) n=3-5 mice per group; (G-H) n=3-4 mice per group. Data shown as mean ± SEM are representative of three independent experiments; *P < 0.05, ***P < 0.001, ****P < 0.0001, ns: not significant, P >0.05; ANOVA with Sidak multiple comparisons test. [file Image_6.tif]

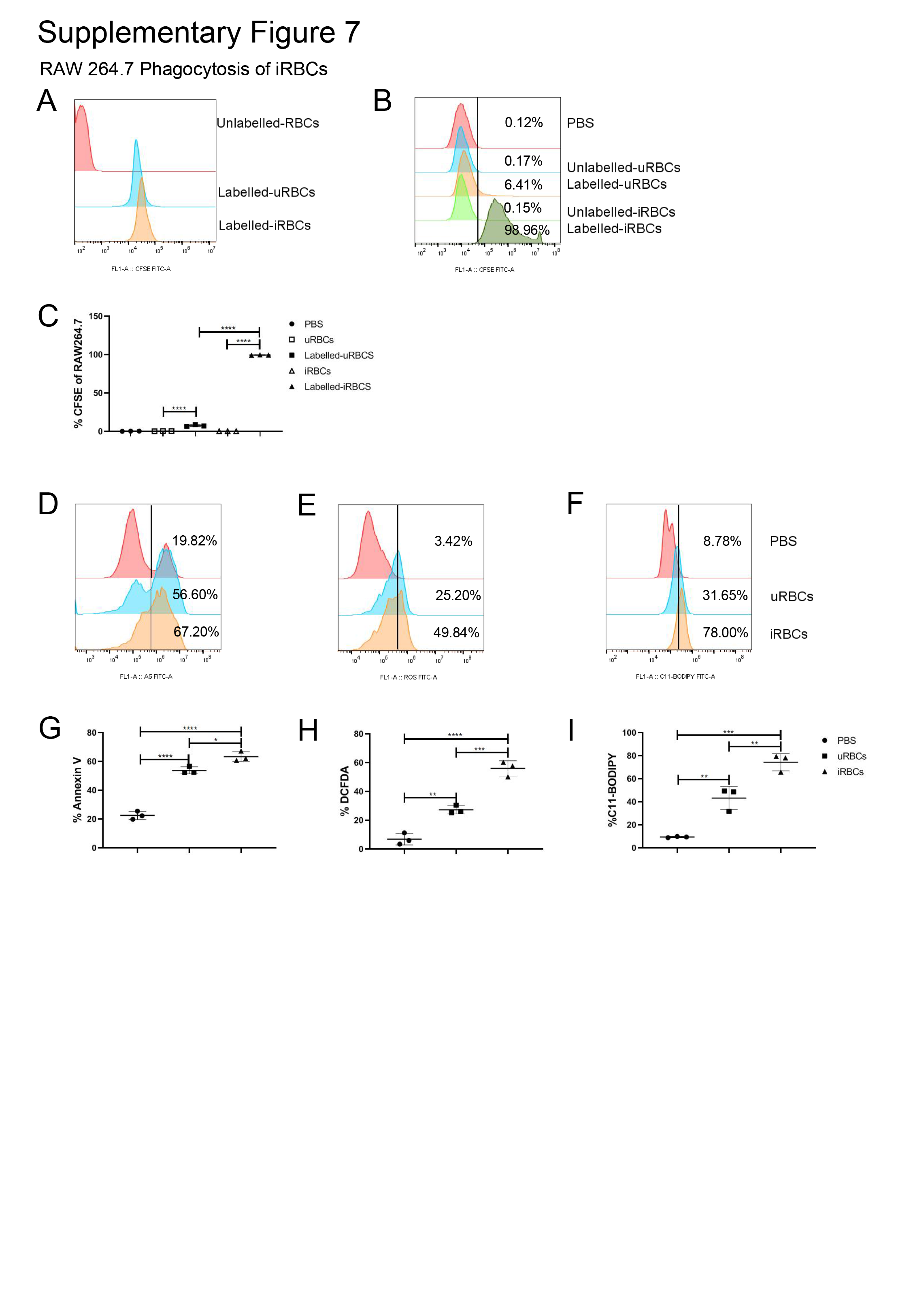

Supplement: Supplementary Figure 7 — RAW264.7 phagocytose iRBCs efficiently in vitro. (A) The representative histogram indicates that uRBCs and iRBCs were both labeled by CellTrace CFSE. RAW264.7 cells were cocultured with unlabeled uRBCs, labeled uRBCs, unlabeled iRBCs, and labeled iRBCs respectively at the ratio of 1:5 for 5 h. (B,C) The percentage of CFSE in RAW264.7 cells was measured and analyzed. (D, G) The percentage of Annexin V+ RAW264.7 cells was measured and analyzed. (E, H) ROS in RAW264.7 cells was measured and analyzed. (F, I) Lipid peroxidation in RAW264.7 cells was measured and analyzed. (C) n=3 samples per group; (G-I) n=3 samples per group. Data shown as mean ± SEM are representative of three independent experiments; *P < 0.05, **P < 0.01,***P < 0.001, ****P < 0.0001, P >0.05; ANOVA with Sidak multiple comparisons test. [file Image_7.tif]

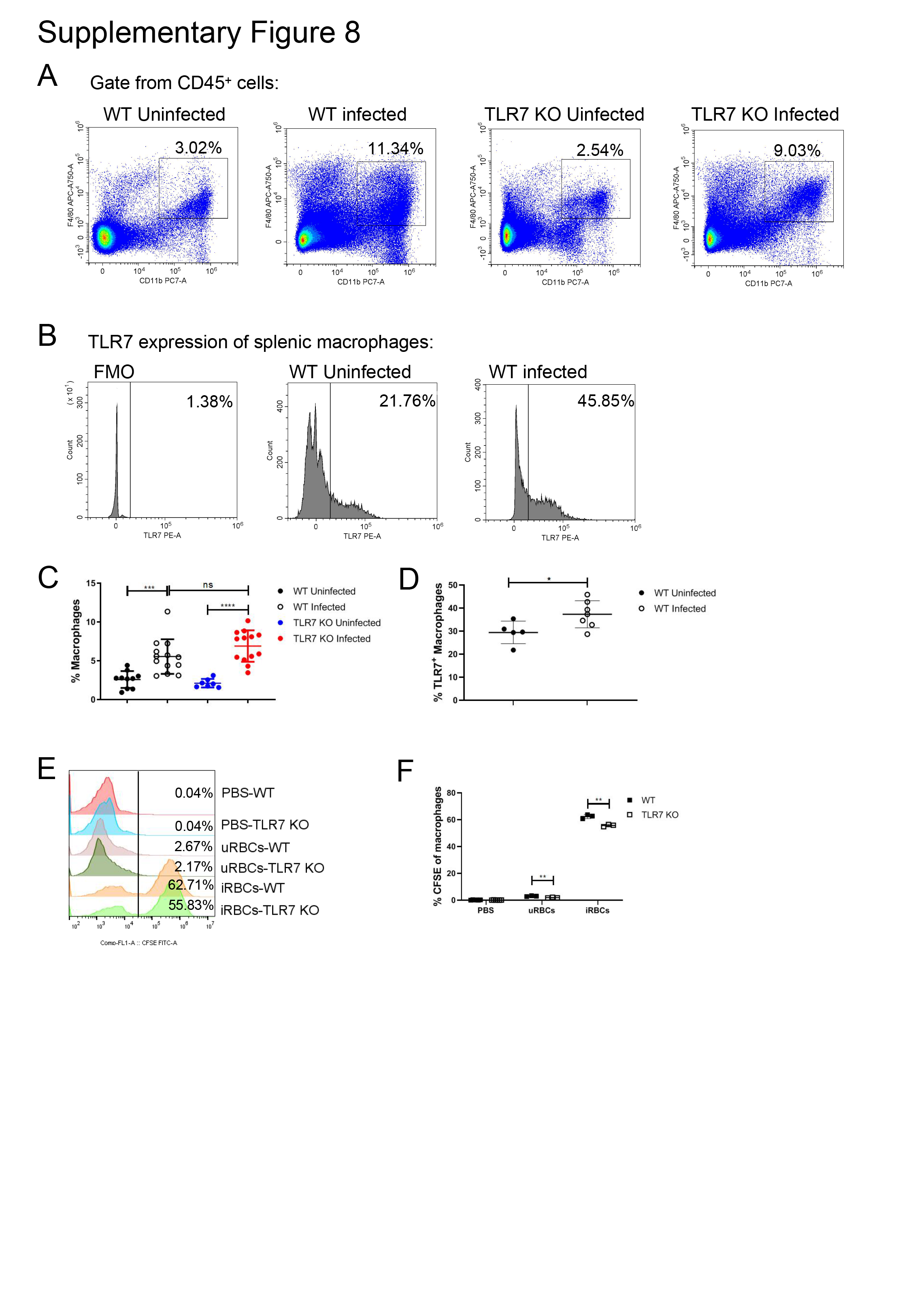

Supplement: Supplementary Figure 8 — The knockout of TLR7 suppresses the phagocytosis of iRBCs by macrophages in vitro. (A) Representative pseudocolor plots of splenic macrophages. (B) Representative histograms of the TLR7 expression in splenic macrophages. (C) The percentage of splenic macrophages in wild-type uninfected and infected, TLR7 -/- uninfected and infected mice were analyzed at 16 dpi. (D) Expression levels of TLR7 in splenic macrophages from wild-type uninfected and infected mice were analyzed at 16 dpi. Splenocytes from wild-type and TLR7 -/- uninfected mice were cocultured with labeled uRBCs or labeled iRBCs at the ratio of 1: 1 for 5 h. (E) The representative histogram shows the percentage of CFSE in macrophages cocultured with uRBCs or iRBCs, and (F) the percentage of CFSE in macrophages was analyzed. (C) n=7-13 mice per group; (D) n=5-7 mice per group; (F) n=3-6 samples per group. Data shown as mean ± SEM are representative of three independent experiments; *P < 0.05, **P < 0.01,***P < 0.001, ****P < 0.0001, ns: not significant, P >0.05; ANOVA with Sidak multiple comparisons test or unpaired t-test. [file Image_8.tif]

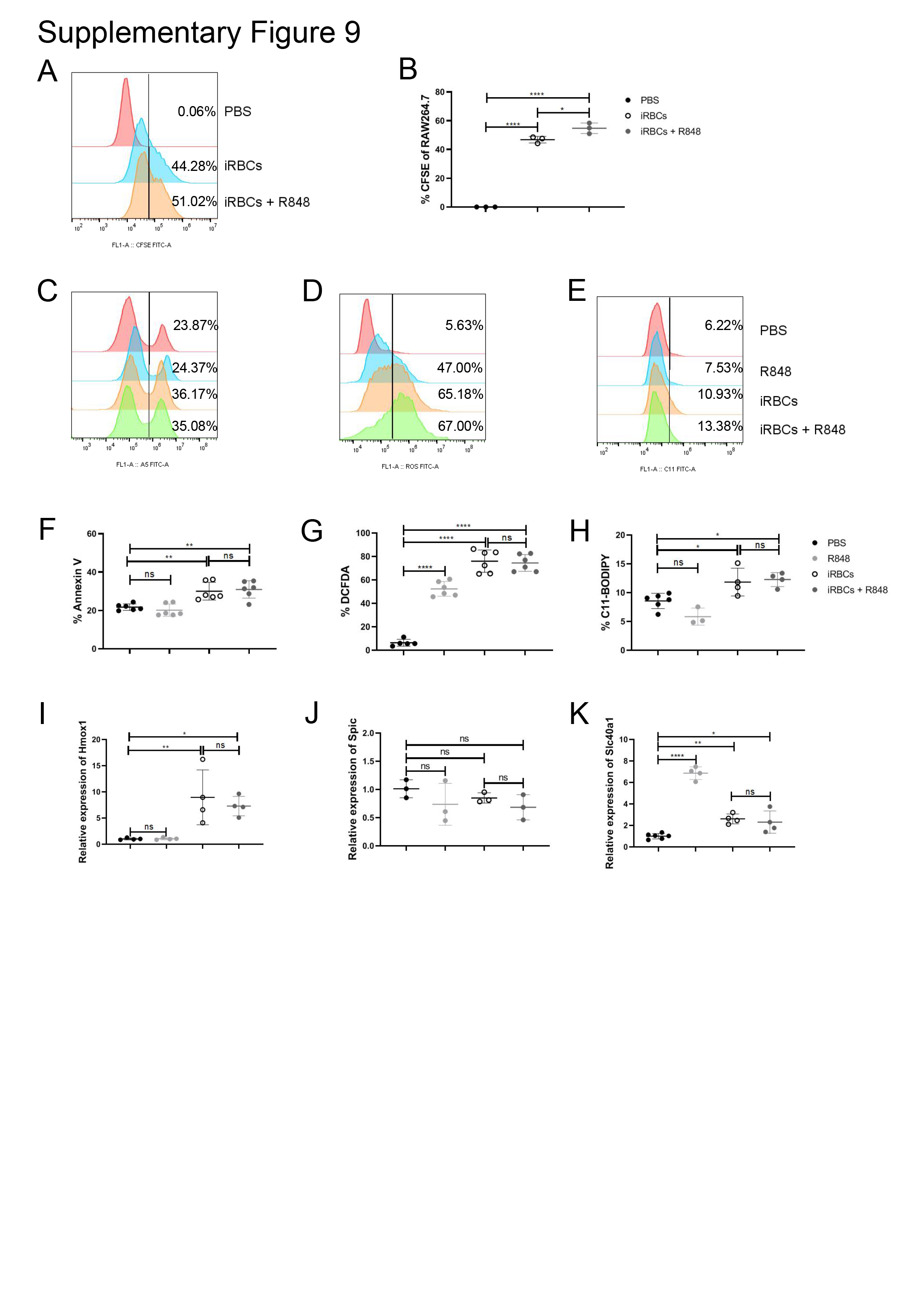

Supplement: Supplementary Figure 9 — R848 augments the RAW264.7 phagocytosis of iRBCs in vitro. RAW264.7 cells were cocultured with labeled iRBCs at the ratio of 1:3 with or without R848 treatment for 5 h. (A) The representative histogram shows the percentage of CFSE in RAW264.7 cells, and (B) the percentage of CFSE in RAW264.7 was analyzed. RAW264.7 cells were stimulated with PBS as a control, or with R848 (2 µg/ml), iRBCs (iRBCs: cells = 3: 1), and R848 plus iRBCs for 20 h respectively. After that, (C, F) The percentage of Annexin V+ RAW264.7 cells was measured and analyzed; (D, G) ROS in RAW264.7 cells was measured and analyzed; (E, H) lipid peroxidation in RAW264.7 cells was measured and analyzed; (I-K) Relative Hmox1, Spic and Slc40a1 mRNA levels in RAW264.7 cells were measured by qPCR respectively. (B) n=3 samples per group; (F-H) n=3-6 samples per group; (I-K) n=3-6 samples per group. Data shown as mean ± SEM are representative of three independent experiments; *P < 0.05, **P < 0.01, ****P < 0.0001, ns: not significant, P >0.05; ANOVA with Sidak multiple comparisons test. [file Image_9.tif]
